# Introduction, spread, and impacts of invasive alien mammal species in Europe

**DOI:** 10.1111/mam.12277

**Published:** 2021-11-23

**Authors:** Lisa Tedeschi, Dino Biancolini, César Capinha, Carlo Rondinini, Franz Essl

**Affiliations:** ^1^ Global Mammal Assessment Programme Department of Biology and Biotechnologies Sapienza University of Rome Viale dell’Università 32 Rome 00185 Italy; ^2^ BioInvasions, Global Change, Macroecology‐Group Department of Botany and Biodiversity Research University of Vienna Rennweg 14 Vienna 1030 Austria; ^3^ Centro de Estudos Geográficos Instituto de Geografia e Ordenamento do Território – IGOT Universidade de Lisboa Rua Branca Edmée Marques, Cidade Universitária 1600‐276 Lisboa Portugal

**Keywords:** biological invasions, environmental impact, Europe, invasive mammals, pathways of introduction, spread, zoonotic diseases

## Abstract

1. Biological invasions have emerged as one of the main drivers of biodiversity change and decline, and numbers of species classed as alien in parts of their ranges are rapidly rising. The European Union established a dedicated regulation to limit the impacts of invasive alien species (IAS), which is focused on the species on a Union List of IAS of particular concern. However, no previous study has specifically addressed the ecology of invasive alien mammals included on the Union List.
2. We performed a systematic review of published literature on these species. We retrieved 262 publications dealing with 16 species, and we complemented these with the most up‐to‐date information extracted from global databases on IAS.
3. We show that most of the study species reached Europe as pets and then escaped from captivity or were intentionally released. On average each year in the period 1981–2020, 1.2 species were recorded for the first time as aliens in European countries, and most species are still expanding their alien ranges by colonising neighbouring territories. France is the most invaded nation, followed by Germany, Italy, and the Russian Federation, and the muskrat *Ondatra zibethicus*, the American mink *Neovison vison*, and the raccoon dog *Nyctereutes procyonoides* are the most widespread species, having invaded at least 27 countries each. Invasive mammals of European Union concern are threatening native biodiversity and human well‐being: worryingly, 81% of the 16 study species are implicated in the epidemiological cycle of zoonotic pathogens.
4. Containing secondary spread to further countries is of paramount importance to avoid the establishment of new populations of invasive mammals and the related impacts on native communities, ecosystem services, and human health.
5. We present a compendium on the ecology and impacts of invasive mammals of European Union concern. It can be used to assist environmental policies, identify and subsequently fill knowledge gaps, and inform stakeholders.

## Graphical abstract

The introduction of a mammal species in few new areas, followed by a lag phase of adaptation and sometimes enriched by further subsequent releases, can rapidly lead to the colonisation of large parts of a continent. On the top left, a heat map with species richness in the countries of Europe. On the top right, a word cloud with the main keywords of our literature search and some of the study species’ names. On the bottom left, four out of 16 study species clockwise from top left: the raccoon dog *Nyctereutes procyonoides*, the muskrat *Ondatra zibethicus*, the racoon *Procyon lotor* and the American mink *Neovison vison*. On the bottom right, the temporal distribution of the first records of the study species in the countries of Europe.

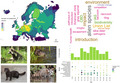

## Introduction

The human‐mediated introduction of species to regions outside their native range has become one of the main drivers of biodiversity change and decline in recent human history (IPBES 2019). Despite a rise in awareness and the adoption of legislation to reduce these introductions, the number of newly introduced species has risen strongly in recent decades (Seebens et al. 2017) and is expected to continue to do so in future (Seebens et al. 2021). International trade, global transportation networks (Hulme 2009), land‐use change (Essl et al. 2020a), and climate change (Diez et al. 2012, Bellard et al. 2018) are the main drivers promoting species’ introduction and spread, and they continue to intensify. Many species introduced in new regions fail to establish self‐sustaining populations or remain localised, whereas others become permanent additions to the receiving ecosystems and spread over substantial distances. In doing so, they can have severe impacts on native biota (Blackburn et al. 2019) at different biological organisation levels (Hawkins et al. 2015), ecosystems services (Vilà & Hulme 2017), and human livelihoods (Bradshaw et al. 2016, Diagne et al. 2021); i.e., they can become invasive alien species (IAS).

The prevention and mitigation of biological invasions in Europe is a significant challenge, as policies are devoted to the free circulation of goods and people (Genovesi et al. 2015). To address this issue, the European Union (EU) adopted the Regulation (EU) No 1143/2014, aimed at the prevention of IAS introduction and spread (EU 2014). The Regulation, informed by years of invasion science research (Genovesi et al. 2015), called for the creation of a list of plant and animal IAS of Union concern, the Union List. Each member state of the EU is required to collect information and take actions related to limiting the introduction and to the detection and eradication of these species, and to mitigate their impact (EU 2014). Furthermore, this subset of IAS is subject to a ban on intentional importation and trade in the EU.

Of the 66 species currently included on the Union List, 11 (~17%) are mammals, highlighting the perceived impact of this taxon across Europe. Indeed, mammals represent 60% of the worst invasive terrestrial vertebrates in Europe (DAISIE 2009, Polaina et al. 2020) and, overall, more than 50 species of alien mammals are currently established in this continent (Biancolini et al. 2021). Alarmingly, due to climate change, suitable climatic space is projected to increase for most invasive mammals in Europe (Polaina et al. 2020). For instance, this is the case for the coypu *Myocastor coypus* (Schertler et al. 2020), the raccoon *Procyon lotor* (Louppe et al. 2019), and the small Indian mongoose *Herpestes auropunctatus* (Louppe et al. 2020). Invasive mammals exert negative impacts on biodiversity through competition (Mazzamuto et al. 2017), disease transmission (Collins et al. 2014), habitat alteration (Nogales et al. 2005), hybridisation (McFarlane et al. 2020), and predation (Dahl & Åhlén 2019).

We provide a comprehensive synthesis of the invasion process, current distribution, and impacts of the invasive mammals of Union concern, by reviewing the literature for these species. Specifically, we: 1) analyse trends in the recently published literature regarding 16 mammal species of Union concern (and candidate species to be included in the Union List that are invasive alien mammals established in Europe and prioritised for 2018–2020) in the last 15 years (2005–2020); 2) summarise pathways of introductions; 3) reconstruct the temporal trajectories of mammal invasions; 4) illustrate geographic distribution patterns; and 5) investigate environmental and 6) social impacts, with a focus on human health. This review updates the current knowledge on a subset of highly impacting mammals. This knowledge is crucial, especially in the light of recent developments in international agreements to protect native biodiversity (EU 2014, CBD 2020). Our review will aid the protection of native biodiversity and informs a wide audience of stakeholders and practitioners.

## Methods

We searched for relevant publications on invasive mammals of EU concern. To provide a wider geographic context, the study area was not limited to the EU; we define as the ‘European territory’ the 47 member states of the Council of Europe, including also the outermost regions of the EU located in the North Atlantic (i.e. Azores, Madeira, and Canary Islands), but excluding the remaining ones (e.g. French Guiana, Guadeloupe). Of the 11 mammal species with self‐sustaining populations included on the Union List, we selected 10 (thus excluding the Eastern fox squirrel *Sciurus niger*, as no established populations are currently present in Europe). Further, based on the work of Carboneras et al. (2018), we selected another six species recommended for future inclusion with high priority (i.e. for the time frame 2018–2020 and with impacts classified as ‘major’ or ‘massive’; see Carboneras et al. 2018). This selection excluded the globally ubiquitous species brown rat *Rattus norvegicus* and species that are not yet found or established in Europe, therefore having no recorded impacts. Finally, a total of 16 established species were included in this review (Table 1).

**Table 1:** The 16 species included in this review. Scientific name, common name, native zoogeographic realms (following Holt et al. 2013), year of first record in Europe, and country of first record in Europe are indicated. Native zoogeographic realms are given for each species in decreasing order, based on the percentage of native range located in each realm

Scientific name	Common name	Native zoogeographic realms	First record	Country of first record
*Axis axis*	Chital	Oriental, Sino‐Japanese	1750	Germany
*Eutamias sibiricus* (Laxmann, 1769)	Siberian chipmunk	Palaearctic, Sino‐Japanese	1850	Russia
*Cervus nippon*	Sika deer	Sino‐Japanese, Palaearctic, Oriental	1860	United Kingdom
*Sciurus carolinensis*	Eastern grey squirrel	Nearctic	1876	United Kingdom
*Myocastor coypus*	Coypu	Neotropical	1882	France
*Muntiacus reevesi*	Reeves’ muntjac	Oriental, Sino‐Japanese, Palaearctic	1894	United Kingdom
*Ondatra zibethicus*	Muskrat	Palaearctic, Nearctic	1905	Czech Republic
*Herpestes auropunctatus* (É. Geoffroy Saint‐Hilaire, 1818)	Small Indian mongoose	Oriental, Saharo‐Arabian, Sino‐Japanese	1910	Croatia
*Neovison vison*	American mink	Palaearctic, Nearctic	1923	Russia
*Nyctereutes procyonoides*	Raccoon dog	Sino‐Japanese, Palaearctic, Oriental	1926	Russia
*Procyon lotor*	Raccoon	Panamanian, Nearctic	1927	Germany
*Castor canadensis*	American beaver	Palaearctic, Nearctic	1935	Finland
*Atlantoxerus getulus*	Barbary ground squirrel	Saharo‐Arabian	1965	Spain
*Callosciurus erythraeus*	Pallas’ squirrel	Oriental, Sino‐Japanese	1974	France
*Callosciurus finlaysonii*	Finlayson’s squirrel	Oriental	1981	Italy
*Nasua nasua*	South American coati	Neotropical	2003	Spain

### Literature search

The literature search was carried out by the first author following the Preferred Reporting Items for Systematic Reviews and Meta‐Analyses (PRISMA) methodology (Appendix S1; Moher et al. 2009) in August and September 2020. For each species, we downloaded available information from the EU Commission CIRCA website (https://circabc.europa.eu/ui/welcome) in the form of the EU Non‐Native Risk Assessment Scheme or the Great Britain Non‐Native Risk Assessment Scheme. In addition, we downloaded CABI species’ datasheets (www.cabi.org) and the NOBANIS factsheets (www.nobanis.org). Hereafter, for brevity, we will refer to all these documents as ‘datasheets’. As these datasheets were highly comprehensive on the scientific knowledge of the study species at the time of completion, the time range of the search for additional publication was adapted for each species, depending on the date of the most recent datasheet. If no prior datasheet was found, the search in the literature databases was performed without a temporal filter.

Subsequently, we searched for additional recent information on each species in Scopus and the Web of Science. In Scopus, we conducted an advanced search refined for the sub‐areas of Agricultural and Biological Sciences and Environmental Sciences. In the Web of Science, we performed a basic search without sub‐area limitations, except for the American beaver *Castor canadensis*, for which we filtered Web of Science results due to the large literature retrieved on unrelated topics (such as engineering or fluid mechanics). For each species, we conducted a separate search with a combination of the scientific name and its synonyms, common name(s), and the relevant keywords, linked by the Boolean operators AND/OR. The list of countries encompassing the alien range, to be used as species‐specific keywords, was obtained from the global Distribution of Alien Mammals database (DAMA; Biancolini et al. 2021). Keywords were identified *a priori* based on the known alien distribution of each species, European Regulation, invasion history, characteristics linked to invasiveness, and impacts caused (Appendix S1).

Two species are identified with different scientific names in the Union List and the International Union for Conservation of Nature (IUCN) Red List, namely the small Indian mongoose (*Herpestes javanicus* in the Union List and *Herpestes auropunctatus* in the Red List) and the Siberian chipmunk (*Tamias sibiricus* in the Union List and *Eutamias sibiricus* in the Red List). We are aware of the recent taxonomic revision, and in this work, we chose to follow the IUCN taxonomy (IUCN 2020).

### Data extraction and preparation

To be included in the review, literature results had to fulfil the following criteria: refer to the European territory (defined as described above), be written in English, and contain information related to at least one of the following: 1) year(s) of first record of a study species, 2) location(s) of first record, 3) pathway(s) of introduction, and 4) impact(s).

The publications we collected were subjected to screening by reading the title and abstract; if these elements did not provide definite information, the full text was screened. After this screening, the full text of each retained publication was assessed for eligibility. The same publication investigating two (or more) focal species was counted for each species, but duplicates were removed for higher‐level analyses. A primary research topic was assigned to each publication, based on its aims, as follows: community ecology, datasheet (sub‐topics: CABI, NOBANIS), ecological modelling, economic impacts, environmental impacts (sub‐topics: competition, disease transmission, habitat alteration, hybridisation, predation), general ecology (sub‐topics: activity pattern, behavioural responses, diet, reproduction, space use), genetics (sub‐topics: genotyping, methodology, phylogeny, population genetics), health status, management, population status, review, risk assessment (sub‐topics: EU Non‐Native Risk Assessment Scheme, Great Britain Non‐Native Risk Assessment Scheme, other), social impacts, and systematics. Publications of pathogens were classified based on whether they were focused on the threats posed to native fauna (topic: environmental impacts/disease transmission), on threats to humans (social impacts), or on general investigation of the invasive species’ pathogens (health status).

In the Tables and Figures, countries are indicated by their International Organization for Standardization country codes, and RU refers to the European part of the Russian Federation. Countries for which information on the study species was not available or was not informative (e.g. Turkey) are not shown in the Figures. Species with occasional occurrences (i.e. not established) or with an unknown status are indicated as ‘casual presences’. Alien geographic ranges for the study species were obtained from DAMA (Biancolini et al. 2021), as well as from the list of all established mammals in Europe, regardless of their inclusion in the Union List, to get a more comprehensive picture of alien mammals’ status in Europe. Native zoogeographic realms (Holt et al. 2013) for the study species and all established mammals in Europe were obtained based on species’ native ranges (IUCN 2020). Marginal parts of native ranges occurring in less than 1% of a zoogeographic realm were not considered.

Capellini et al. (2015) and Blackburn et al. (2017) identified body size, litter size, litters per year, and generation length as species’ traits affecting the introduction, establishment, and spread of invasive mammals. We extracted these trait values from the recently developed Coalesced Mammal Database of Intrinsic and Extrinsic traits (COMBINE; Soria et al. 2021). Reproductive life span was calculated as the difference between maximum longevity and age at first reproduction (Soria et al. 2021).

Each species was assigned to one or more pathway(s) of introduction following Convention on Biological Diversity (CBD) categorisation (CBD 2014, Biancolini et al. 2021). First records of the species were mainly obtained from version 2 (last updated in March 2021) of the Alien Species First Records Database (Seebens et al. 2017). For first records obtained from publications encountered during the literature review, the earliest year was retained in cases of multiple introductions or continuous introduction into a country. Information regarding species’ pathogens (e.g. prevalence) was extracted both from original publications and from reviews encountered during the literature search.

## Results

### Literature search

The literature search yielded 3322 publications published between 2005 and 2020. All the species but one (Barbary ground squirrel *Atlantoxerus getulus*) had at least one datasheet available for download, resulting in a total of 36 published datasheets. After the first screening, 591 publications (not including the datasheets) were retained. A backward reference search (‘snowballing’) was performed on the reference list of each of these publications to identify other relevant publications, adding a further 30 publications. Duplicate records resulting from an overlap of the database outcomes were removed. 26 publications could not be assessed due to access restrictions. Eventually, 262 publications were included in the review (Appendix S2).

Published information was available mostly for the raccoon (that accounted for 16% of all publications), the American mink (14%), and the sika deer *Cervus nippon* (12%; Appendix S3). The majority of the datasheets collected (88%) were published from 2009 to 2014 (Appendix S3) and, due to the temporal filters adopted, for most of the study species the literature search supplied mainly publications issued after 2015. Accounting for these filters adopted in the literature search, mainly species’ environmental impacts were investigated (24% of all publications), with a peak of publications in 2017‐2018, followed by publications on health‐related issues (18%) and social impacts (12%).

### Taxonomic characterisation, traits, and native ranges

The 16 study species belong to three orders and nine families. Half of them belong to the order Rodentia (Appendix S3); the remaining species are either from Carnivora (31%) or from Artiodactyla (19%). The Sciuridae family is the most represented, accounting for 31% of all species, followed by Cervidae (19%) and Procyonidae (13%). In comparison, the full ensemble of 53 alien mammal species established in Europe is divided into seven orders and 17 families (Biancolini et al. 2021). The order Artiodactyla is the most numerous (28%), followed by Rodentia (23%) and Carnivora (17%; Appendix S3). The most represented family is Cervidae (15%), followed by Leporidae (13%) and Bovidae and Mustelidae (11% each).

Adult body mass for the study species varied between 85 g for the Siberian chipmunk and 53000 g for the sika deer (mean for all study species 9762 g, median 2499 g, interquartile range 8305 g; Appendix S3). Litter size was between one for sika deer and Reeves’ muntjac *Muntiacus reevesi* and 6.4 for the muskrat (mean for all 16 species: 3.3 young per litter). Litters per year ranged from one for American beaver, sika, American mink, and raccoon to 2.6 for the muskrat (mean for all species: 1.5 litters per year). Lastly, generation length (in days) ranged between 2941 for the Barbary ground squirrel and 8504 for the sika deer (mean for all species: 5781 days).

The study species originate mainly from the Palaearctic, Sino‐Japanese, and Oriental zoogeographic realms (Appendix S3). Similarly, the full ensemble of alien mammals established in Europe originates mainly from the Palaearctic, Saharo‐Arabian, and Sino‐Japanese realms (Appendix S3).

### Pathways of introduction to Europe

**Fig. 1:**
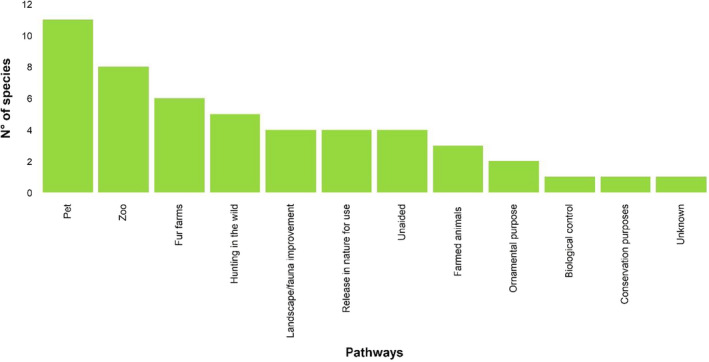
Convention on Biological Diversity’s pathways of introduction applied to the study species in Europe (*n* = 16). Each species was assigned to one or more pathways (*n* = 50). Pathways with zero occurrences or nomenclature not relevant for terrestrial mammals are not shown. Pathway names are abbreviated following CBD (2014).

The main pathway of introduction for the study species in Europe was the pet trade (Fig. 1): 69% of the species escaped after they were introduced at least once through the pet trade (i.e. from private owners), 50% escaped from zoos (i.e. from public exhibitions), and 38% escaped after they were introduced to be bred in fur farms. One species was released in nature for biological control (the small Indian mongoose in Croatia), and another one for conservation purposes (the American beaver in Finland). The chital *Axis axis*, introduced in Croatia, was the only species with an unknown introduction pathway, although subsequent repeated introductions within Croatia were reported for hunting purposes (Šprem & Zachos 2020). No study species was reported to be introduced as a contaminant, as a stowaway, or via a corridor.

### Temporal trajectories of mammal invasions in Europe

**Fig. 2:**
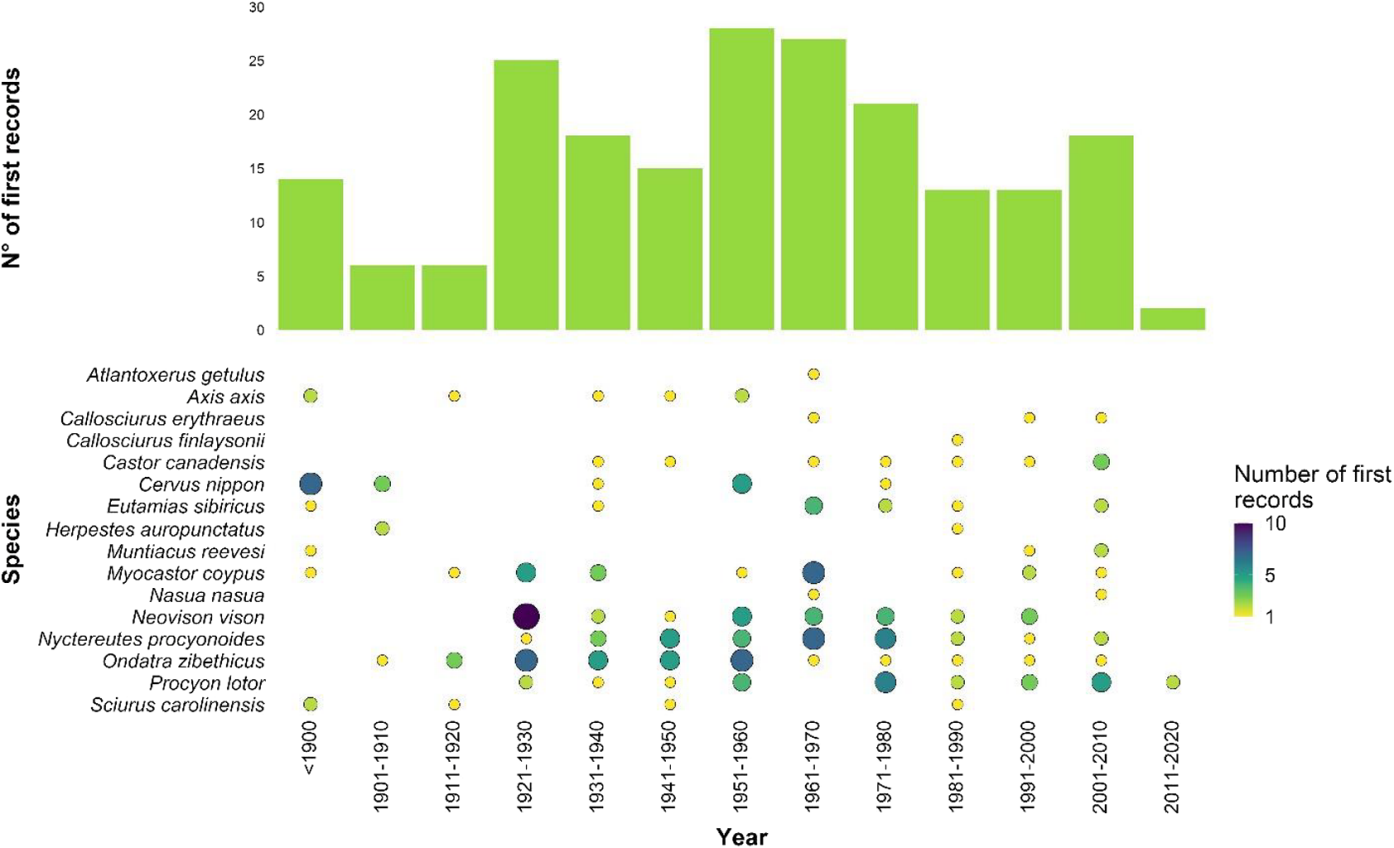
Temporal distribution of first records (*n* = 197) of the study species in the countries of Europe. Point sizes represent the number of records per species and time period.

The rate of first records (of both established and casual presence records) of the study species in countries of Europe increased on average from 1.4 new records/year over a 40‐year period (1900‐1940) to 2.3 records/year in 1941‐1980, and then dropped to 1.2 records/year in 1981‐2020 (Fig. 2, Appendix S4). Overall, the American mink, the raccoon dog, and the muskrat accounted together for 47% of first records.

### Geographic distribution patterns in Europe

The UK and the Russian Federation first recorded three of the study species each, namely the sika deer (1860), the Eastern grey squirrel *Sciurus carolinensis* (1876), and Reeves’ muntjac (1894) for the UK, and the Siberian chipmunk (1850), the American mink (1923), and the raccoon dog (1926) for the Russian Federation (Appendix S4).

**Fig. 3:**
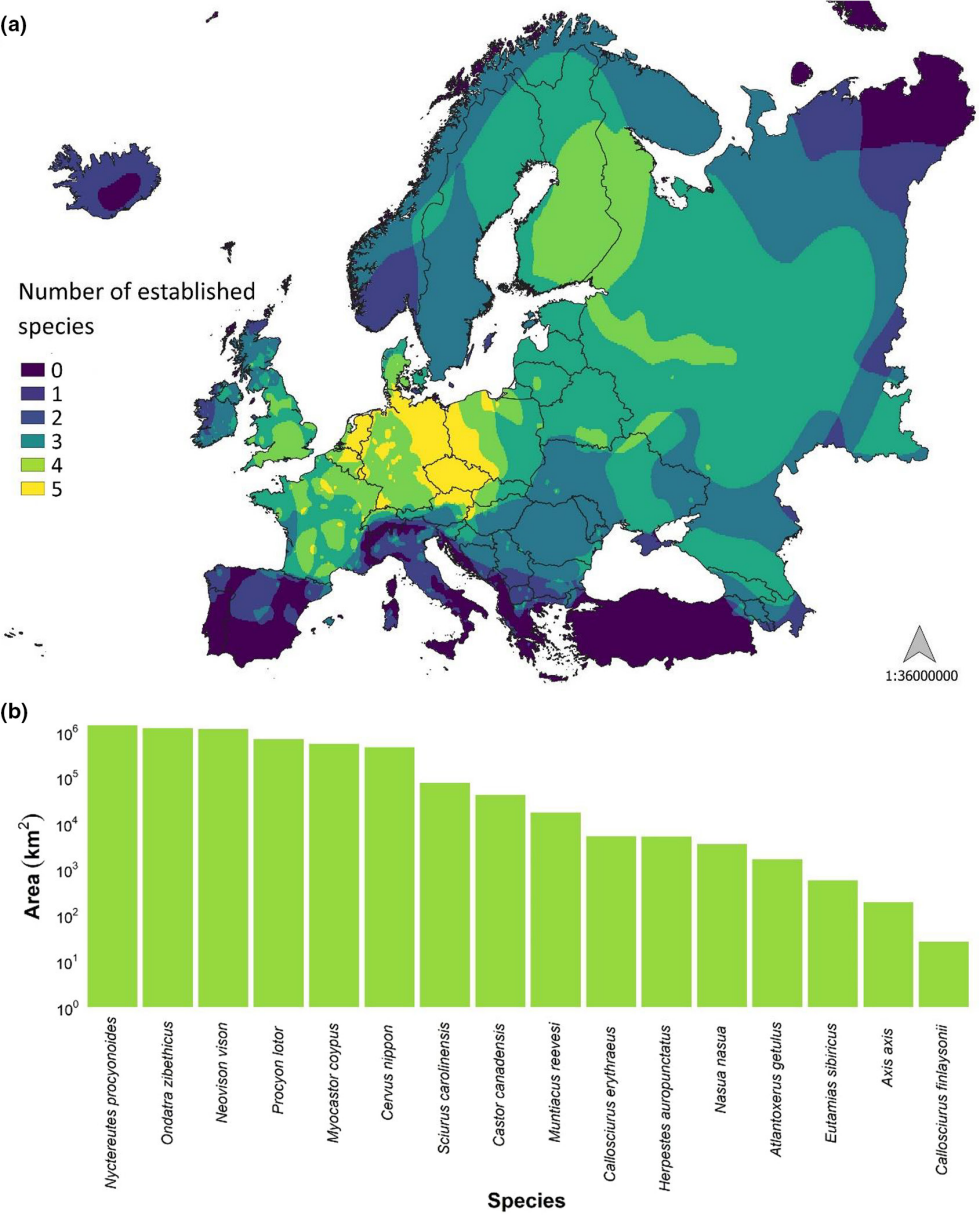
Established presence of the study species in Europe: (a) heat map showing study species richness in the study area (alien range maps source: Biancolini et al. 2021); (b) area (log scale, km^2^) occupied by the study species.

Considering the number of countries occupied, the most widespread species was the muskrat (established in 32 countries and with casual presence records in three countries; Appendix S3), followed by the American mink (established in 28, casual in seven), the raccoon dog (established in 27, casual in seven), and the coypu (established in 24, casual in four). However, with respect to the area occupied only by the established species, the order slightly changes, with the raccoon dog becoming the most widespread, followed by the muskrat, the American mink, and the raccoon (Fig. 3).

**Fig. 4:**
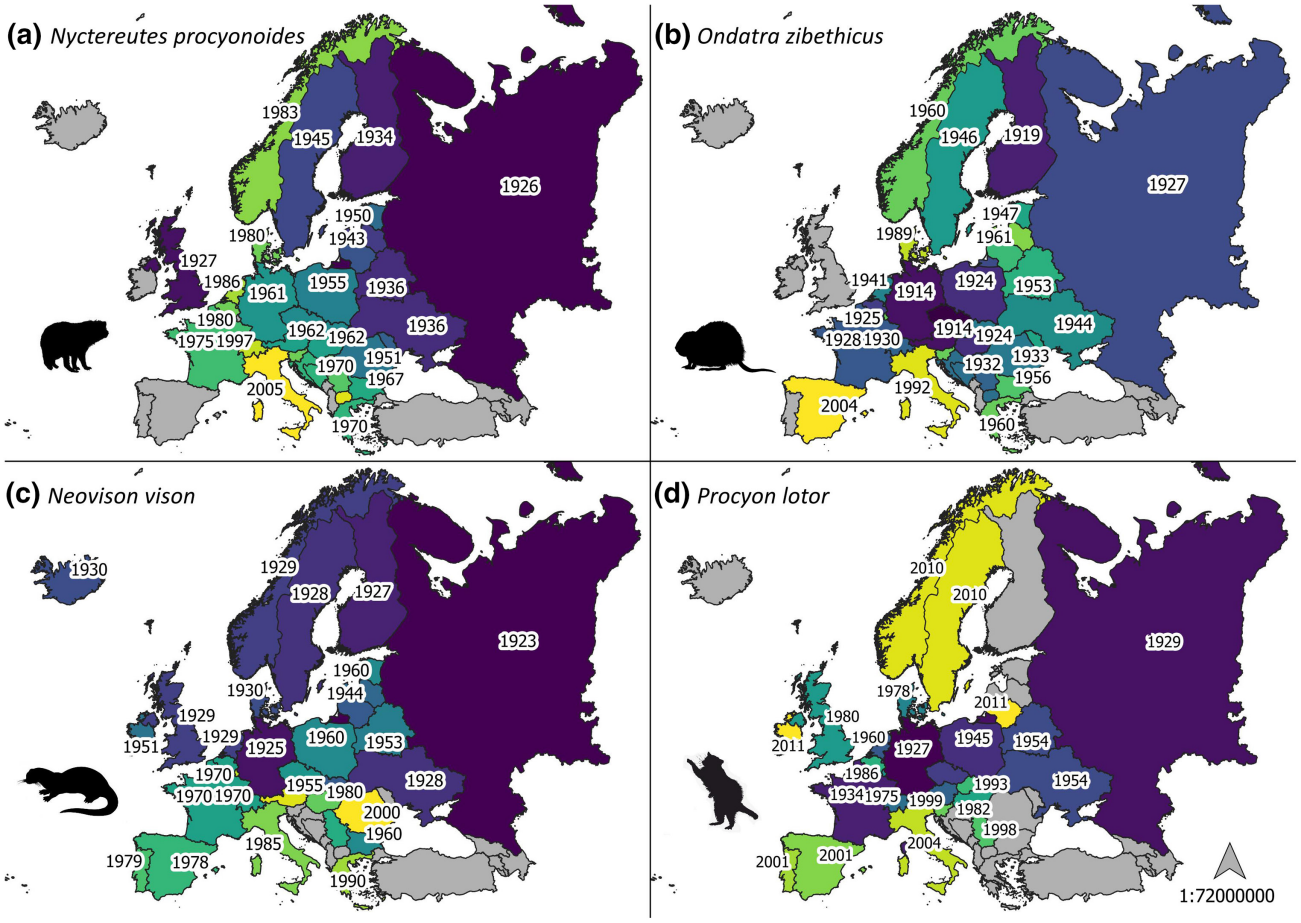
Spread trajectories of the four species that invaded most of the European territory: (a) the raccoon dog, (b) the muskrat, (c) the American mink, and (d) the raccoon. Countries are graded from the country invaded earliest (darker) to the latest (lighter). Year of the first record (when available) is shown. Countries without the presence (established or casual) of the species are shown in grey. [Correction added on 9 February 2022, after first online publication: Figure 4 has been replaced.]

Fig. 4 illustrates the invasion waves of the four species that invaded most of the European territory (the raccoon dog, the muskrat, the American mink, and the raccoon), including established presence and casual records.

### Environmental and socio‐economic impacts in Europe

Among the publications retrieved during this search process, environmental impacts of invasive mammals have been broadly investigated, as shown by the number of publications issued on this topic (*n* = 63; Appendix S3). Disease transmission was the most studied sub‐topic (30% of the total number of publications related to environmental impacts), followed by predation (24%) and competition (19%). Publications on pathogens of invasive mammals (*n* = 19) revolved mainly around their helminthofauna (51% of the publications on pathogens; Appendix S3). Some pathogens were introduced in Europe with the study species, such as the nematode *Strongyloides callosciureus*, introduced with Pallas’ squirrel *Callosciurus erythraeus* and potentially infecting the native Eurasian red squirrel *Sciurus vulgaris* due to spill‐back and spill‐over processes (Mazzamuto et al. 2016); and the squirrelpox virus, which can be lethal for red squirrels and was introduced in the UK and Ireland with the Eastern grey squirrel (IUCN 2005, Invasive Species Ireland 2012).

**Fig. 5:**
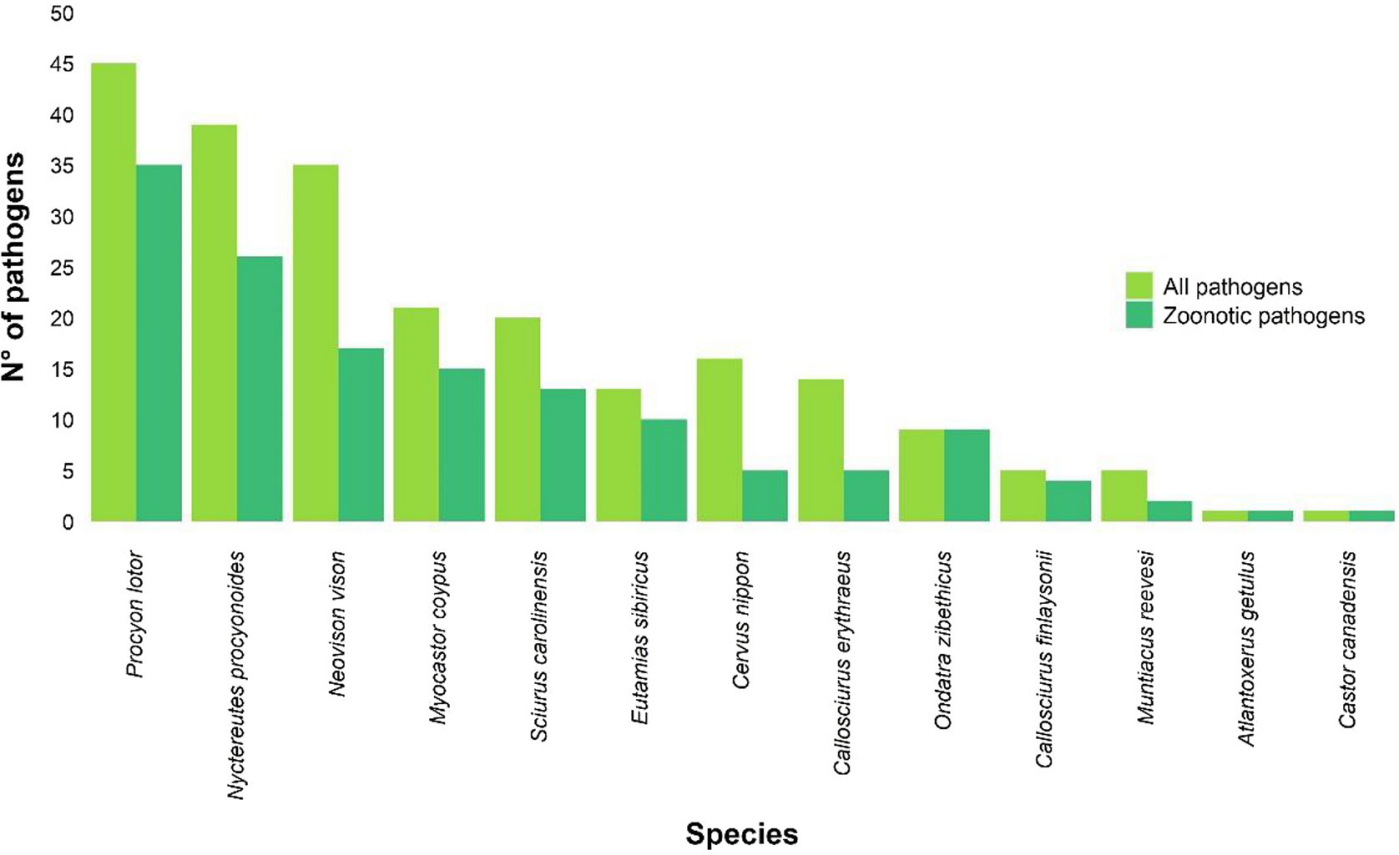
Total number of pathogens known to infect the study species (all pathogens) and pathogens with zoonotic potential (zoonotic pathogens). Species without recorded pathogen infections are not shown.

The study species were found to be infected by 224 pathogens, of which 143 (64%) have zoonotic potential; 13 study species serve as potential reservoirs or are implicated in their epidemiological cycle (Fig. 5; Appendix S5). Specifically, regarding the most widespread study species, 49% of the pathogens known to infect the American mink have zoonotic potential; the percentage rises to 67% for the raccoon dog, 78% for the raccoon, and 100% for the muskrat (Fig. 5). Overall, publications on *Echinococcus multilocularis* (14 publications, three species), *Toxoplasma gondii* (nine publications, six species), and *Baylisascaris procyonis* (nine publications, one species) were particularly abundant among the study species (Appendix S5). Prevalence rates presented a high geographic and taxonomical variability: the prevalence of *Echinococcus multilocularis* ranged between 0% (in the raccoon and the raccoon dog in various countries; Kornyushin et al. 2011, Wahlström et al. 2012, EFSA 2015, Karamon et al. 2016, Oksanen et al. 2016, Duscher et al. 2017) and 28% (in the racoon dog in Slovakia; Oksanen et al. 2016); for *Toxoplasma gondii*, it ranged from 0% (in American mink in Spain and the raccoon in the Czech Republic; Criado‐Fornelio et al. 2018, Kornacka et al. 2018) to 79% (in American mink in Spain; Ribas et al. 2018); lastly, the prevalence of *Baylisascaris procyonis* in raccoons ranged from 2% in Poland (Karamon et al. 2014) to 80% in Germany (Hohmann et al. 2002). There are recent reports from Denmark and the Netherlands of SARS‐CoV‐2 infection in mink (Oreshkova et al. 2020).

Regarding the second most investigated sub‐topic (predation; *n* = 15), the majority of publications analysed the predatory effects of American mink (40% of the total number of publications related to predation), raccoon dogs (27%), and Eastern grey squirrels (20%). Of the publications of predation by American mink, 67% were performed in Poland; all publications of predation by raccoon dogs were performed in Scandinavia, and 67% of publications of predation by Eastern grey squirrels were conducted in the UK. Lastly, regarding competition (*n* = 12), 75% of the publications investigated how alien squirrels compete with native squirrels. The remaining publications dealing with environmental impacts analysed the sub‐topics of habitat alteration (*n* = 9) and hybridisation (*n* = 8, comprising only publications on sika deer).

Almost a third of the publications dealt with the health status of the species (*n* = 46) or with socio‐economic impact topics (*n* = 31), this latter comprising only socio‐economic impacts connected to human health. Reviews, datasheets, and risk assessments accounted for 21% of the total publications (*n* = 54). The remaining publications investigated general ecology of the species (*n* = 21), genetics aspects (*n* = 17), population status (*n* = 13), community ecology (*n* = 10), ecological modelling (*n* = 9), management (*n* = 3), and systematics (*n* = 3). The least investigated topic was economic impacts (*n* = 1).

## Discussion

The majority of invasive mammals of European Union concern reached Europe as pets that escaped from captivity or were intentionally released. Although introductions of alien mammals have declined in Europe for more than 50 years, many study species are still expanding their alien ranges by colonising neighbouring countries. France is the most invaded country with regard to established presence records, followed by Germany, Italy, and the Russian Federation, and the muskrat, the American mink, the raccoon dog, and the raccoon are the most widespread species. Invasive mammals of Union concern are threatening native biodiversity and human health, and have consequences that were largely overlooked in the past, such as new roles in epidemiological cycles of zoonotic pathogens (Oreshkova et al. 2020).

### Literature search

Geographic and impact‐related biases emerged from the reviewed literature. Charismatic, widespread, and detrimental species received more attention – in terms of publication numbers – than others, a trend already observed in invasion ecology (Pyšek et al. 2008). Apparently, the documented environmental or social impacts and the geographic range size of the alien species are also related to the number of publications. For instance, species that have localised alien distributions, such as island invaders (the Barbary ground squirrel, the chital, the small Indian mongoose, and the South American coati *Nasua nasua*), have been less well investigated than more widespread species, such as the raccoon and the coypu. The well‐acknowledged invasive potential of these localised species urgently calls for additional studies on their impacts and possible future spread. For example, the small Indian mongoose, a devastating island invader globally, could irremediably harm native biota in the Balkans mainland (Ćirović & Toholj 2016).

Our conclusions regarding the recently published literature should be interpreted with caution, as our search did not include ‘grey’ literature or publications published in languages other than English; this may have generated biases and led to apparent knowledge gaps (Angulo et al. 2021).

### Taxonomic characterisation, traits, and native ranges

Humans pose an initial ‘filter’ to species introduction (Clout & Russell 2008) by selecting mammal species with key traits, such as a large body mass, long reproductive life span, and large litter size (Blackburn et al. 2017). These last two key traits have been shown also to promote the subsequent phases of establishment and spread, along with many litters per year (Capellini et al. 2015), intraspecific variation in body traits, native range size, and propagule pressure (González‐Suárez et al. 2015). The mean adult body mass for our species was high – especially if compared with the mean adult body mass for mammals – but 75% of the study species did not weigh much, and a few of them (i.e. sika deer and chital) heavily skewed the mean. Regarding litter size, the most widespread species in Europe (in terms of both countries and area occupied) had also an above‐average litter size, confirming the importance of this trait in the invasion stages consecutive to introduction (Capellini et al. 2015). As for the litters per year, the species that were above average (more than 1.5 litters per year) were mainly rodents. Accordingly, litter size is larger in these socially monogamous species (West & Capellini 2016). Although a longer reproductive life span promotes introduction and establishment in mammals, the study species with a higher value for this trait have rather localised distributions (the sika deer, the Eastern grey squirrel, and Reeves’ muntjac), possibly as an outcome of a low propagule pressure. On the contrary, widespread species (e.g. the muskrat and the American mink) have a short reproductive life span. The discordance of some study species’ traits (adult body mass and reproductive life span) with what was found previously in the literature could be the result of the relative over‐representation in the past of mammals introduced for goods and services (hunting, fur farming, transport; Blackburn et al. 2017), in contrast with more recent introductions of species used as pets (such as squirrels) or for other aesthetic purposes.

With regard to the provenance of the study species, the Palaearctic, Sino‐Japanese, and Oriental realms were equally relevant. Previous studies (Genovesi et al. 2009, 2012) showed that the Palaearctic and the Nearctic were the realms harbouring the native ranges of many introduced mammals. Similarly as for species’ traits, the difference could be linked to the over‐representation in the past of species introduced to be utilised by humans for goods and services. Contrarily, our study species are mostly used as pets and originate from eastern realms.

### Pathways of introduction to Europe

Overall, the study species were mainly kept in private or public collections or bred for fur, and subsequently escaped or were released. We showed that the pet trade was an important pathway of introduction to Europe in the last 15 years. For instance, the Siberian chipmunk was first recorded in Ireland in 2007, and was probably released in nature by (or escaped from) private owners (Invasive Species Ireland 2019). Indeed, all the Sciuridae have been introduced at least once for companionship, enjoyment, recreation, or trading. These species are charismatic and have often been released for ‘fauna improvement’ in urban parks (as in the case of Siberian chipmunks in Italy; Mori et al. 2018).

Higher rates of establishment and spread are related to multiple releases and, in general, to a higher introduction effort (Clout & Russell 2008, Capellini et al. 2015). However, in the absence of accurate introduction records it is often challenging to distinguish between the natural spread of a species from invasion foci in adjacent countries and a deliberate release (for instance, by private owners) or an escape, especially for highly vagile species such as ungulates and carnivores. For example, recent genetic analyses have shown that new Eastern grey squirrel populations in Italy (which supposedly originated from natural dispersal of individuals) derived in fact from other populations established almost 200 km away (Signorile et al. 2016). Therefore, even in the absence of clear evidence of unaided dispersal, it is inappropriate to assign the unaided pathway of introduction to some species (Pergl et al. 2020).

### Temporal trajectories of mammal invasions in Europe

Despite the continuous geographic range expansion of alien mammal species throughout Europe, the numbers of first records of alien mammals declined from the 1960s onwards (Fig. 2). This pattern has already been recorded at a global level for this taxon, and it is likely to be influenced by the most recent first records (Seebens et al. 2017). For instance, there were almost no first records of the study species in the last 10 years. However, longer monitoring is needed to assess the reliability of these trends (Seebens et al. 2017), especially to clarify whether saturation has been finally reached or whether these patterns depend on other factors. The rapid decline in new introduction events can be attributed to the synergistic effects of increased awareness and stricter regulations on alien mammals bred for fur, exploited as game species, or used as pets throughout Europe (Seebens et al. 2017), especially since the implementation of the EU IAS Regulation (EU 2014).

First records in Europe were not evenly distributed among countries, as the UK and the Russian Federation first recorded three study species each. Two of the most common study species (the American mink and the raccoon dog) were first recorded in the Russian Federation, where they were introduced for fur farming. This comes as no surprise, as this country was one of the world’s largest producers and consumers of fur (Balakirev & Tinaeva 2001), although now the production has significantly declined (Khusainova & Vorozheykina 2019).

### Geographic distribution patterns in Europe

In general, the introduction of a species in a few localities, and subsequent further releases, can rapidly lead to the colonisation of large parts of the European territory. We show that, in Europe, the raccoon dog, the muskrat, the American mink, and the raccoon are the most widespread species (in terms of area occupied with established presence), having invaded at least 19 countries each and being present for at least 90 years in the European territory (the most recent invader was the raccoon, introduced in 1927 in Germany). The wide distribution of these species can be attributed to several factors, including their adaptability, capacity to colonise different environments (Birnbaum 2013), wide trophic niches (Bartoszewicz 2011), and high reproduction potentials (Pitra et al. 2010).

It is of paramount importance to monitor the secondary spread (Essl et al. 2020b) of these species in the European territory and to prevent the establishment of new populations of invasive mammals. Secondary spread would foster geographic range expansion for invasive species and would counteract the stringent regulations adopted hitherto to prevent new introductions (and to mitigate IAS impacts). In the EU, changes in the main drivers of potential impacts of biological invasions (trade and transport, climate change, and socio‐economics; Essl et al. 2020a) are ongoing (Kovats et al. 2014). This, combined with free circulation of goods and people within the EU (Genovesi et al. 2015), may promote a rise of impacts of IAS.

### Environmental and socio‐economic impacts in Europe

The wide distribution of alien mammals in the European territory raises many concerns, as these species can transmit diseases to native species, act as disease reservoirs, and introduce zoonotic pathogens. The latter can be hosted by the majority of the study species, and worryingly, some widespread species carry many zoonotic pathogens. Associated infectious diseases, such as echinococcosis, toxoplasmosis, and baylisascariasis, may pose a serious threat to human health. For comparison, only 11% of species on the IUCN list of the 100 World’s Worst Invasive Alien Species are known reservoirs for zoonotic pathogens (Vilà et al. 2021).

Publications on *Echinococcus multilocularis* (the pathogen most commonly analysed in all the publications on disease transmission) revolved mainly around the raccoon dog, as it is the definitive host (the host in which the parasite attains sexual maturity; Bagrade et al. 2016). However, the muskrat is an intermediate host (a host in which a parasite passes one or more of its asexual stages), and only two studies (out of 14) investigated the prevalence of the pathogen in this rodent. Dedicated health surveillance, in general of these widespread species of invasive mammals, would be beneficial for many people, as the study species are often found in cities or are bred in captivity for commercial purposes.

In this context, the outbreaks of SARS‐CoV‐2 reported in the Netherlands and in Denmark in 2020 (Molenaar et al. 2020, Oreshkova et al. 2020) are notable. It is currently unknown which role American mink and other mammals (especially free‐ranging ones that are regularly in contact with humans, such as stray cats and their prey) may play in the SARS‐CoV‐2 cycle. They may act as wild reservoirs or spread new strains of the virus (mutations affecting the spike protein have already been found in American mink; Molenaar et al. 2020, Oreshkova et al. 2020, WHO 2020). American mink appear to be very susceptible to the virus, and cases are being reported from other countries such as Spain, Sweden, Italy, and the USA. Following the huge outbreaks of SARS‐CoV‐2, the mink fur industry in the Netherlands and in Sweden was terminated in 2021 (Humane Society International 2020, 2021), while Italy and Denmark suspended American mink fur farm activity until the end of 2021 (DW 2020, Ministero della Salute 2021).

Large‐scale studies investigating the prevalence of zoonotic and non‐zoonotic pathogens and the possible roles of invasive mammals of Union concern in their epidemiological cycles are still largely missing. The spread of many pathogens follows invasion stages similar to those described for invasive animals and plants (Vilà et al. 2021), and the unknown role of alien mammals as reservoirs in the wild could easily jeopardise the efforts in place to prevent, manage, or eradicate zoonotic diseases. Due to the many analogies between invasion science and human emerging infectious diseases, lessons from the management of IAS can be applied to tackling future human epidemics (Vilà et al. 2021).

Despite their key role in disease epidemiology, predation is probably the most well‐known mechanism through which alien mammals can imperil native biodiversity. Through predation, the American mink can exert a negative effect on species such as the Eurasian water vole *Arvicola amphibius* (Rushton et al. 2000, Mori & Mazza 2019) and threaten genetically distinct populations of prey species (Flávio et al. 2021). Heavier egg predation on ground‐nesting birds (compared with previous studies) has recently been reported for the raccoon dog (Dahl & Åhlén 2019), and the muskrat was found to be a major threat to endangered freshwater bivalves in Germany (Stoeckl et al. 2020).

Besides predation and disease transmission, invasive mammals can contribute to native species’ extinction through other mechanisms, such as competition (Bertolino & Lurz 2013). The Eurasian red squirrel went extinct in more than half of its range in Italy and was replaced by the Eastern grey squirrel (Bertolino et al. 2016), while the American mink colonised the area occupied by the European mink *Mustela lutreola* and confined this Critically Endangered native mustelid to few areas in Spain (Põdra & Gómez 2018).

Invasive alien species can exert a plethora of different impacts on human well‐being (e.g. on personal safety, material and immaterial assets, or cultural relations; Bacher et al. 2017). However, the only type of socio‐economic impact that emerged prominently from the literature we reviewed was the negative impact on human health. Information regarding economic impacts was not abundant in our results. This is likely to be due to the problems linked to economic data collection (Bradshaw et al. 2016), the specificity of economic sectors (Paini et al. 2016), difficulties in monetising economic damages of IAS (Diagne et al. 2020), or the restricted spatial scales of most studies (Hoffmann & Broadhurst 2016). Until recently, the only exhaustive inventory of economic costs associated with IAS existed solely for insects (Bradshaw et al. 2016, Diagne et al. 2020). Although the InvaCost database (Diagne et al. 2020) is now the most updated datasource for this type of information, the data contained within it for our study species were insufficient for the purpose of this review.

## Conclusions

In the European territory, the muskrat, the American mink, and the raccoon dog are the most widespread invasive mammal species, and France, Germany, Italy, and the Russian Federation are the most invaded countries. The 16 species of invasive mammals of European Union concern are threatening native biodiversity and human well‐being. The pet trade is still the main pathway of introduction for alien mammals into Europe. It is currently unclear whether the recent decline in first records is due to the stricter measures adopted by the European Union or whether it is the result of a saturation effect. To explain this decline, longer and more accurate monitoring of first records and of secondary spread of the invasive mammals of Union concern is necessary.

The eradication of those study species with wide distributions is unlikely to be feasible. However, alien species themselves are neither ‘bad’ nor ‘good’: it is rather populations of the species that have become invasive, that can be problematic (Simberloff et al. 2013), and that should be managed. In this context, the identification of problematic populations or invaded areas may help to mitigate future impacts.

## Funding

The authors appreciate funding by the Sapienza University of Rome, the 2017‐2018 Belmont Forum and BiodivERsA joint call for research proposals, under the BiodivScen ERA‐Net COFUND programme, and with the funding organisations FWF (AlienScenarios, FWF project no I 4011‐B32), and the Portuguese National Funds through Fundação para a Ciência e a Tecnologia (CEECIND/02037/2017; UIDB/00295/2020 and UIDP/00295/2020). Open Access Funding provided by Universita degli Studi di Roma La Sapienza within the CRUI‐CARE Agreement.

[Correction added on 3 June 2022, after first online publication: CRUI funding statement has been added.]

## Supporting information


**Appendix S1.** Process of literature search and keywords used.

**Appendix S2.** List of the publications obtained through the literature search process for each study species in Europe.

**Appendix S3.** Figures illustrating the trends in the published literature, species’ taxonomy, traits, native zoogeographic realms, and pathogen classification.

**Appendix S4.** Year of first record and presence records for the study species in Europe.

**Appendix S5.** List of pathogens known to have been recorded to infect the study species in Europe and list of additional references.
