# Supplementary material for: Introduction, spread, and impacts of invasive alien mammal species in Europe
Source: Mamm Rev. 2021 Nov 23;52(2):252–66. doi: 10.1111/mam.12277 (PMC9299096; doi:10.1111/mam.12277)
Supplement: Supplementary file 1 — Appendix S1. Process of literature search and keywords used. [file MAM-52-252-s002.docx]

**Appendix S1.** Process of literature search and keywords used.


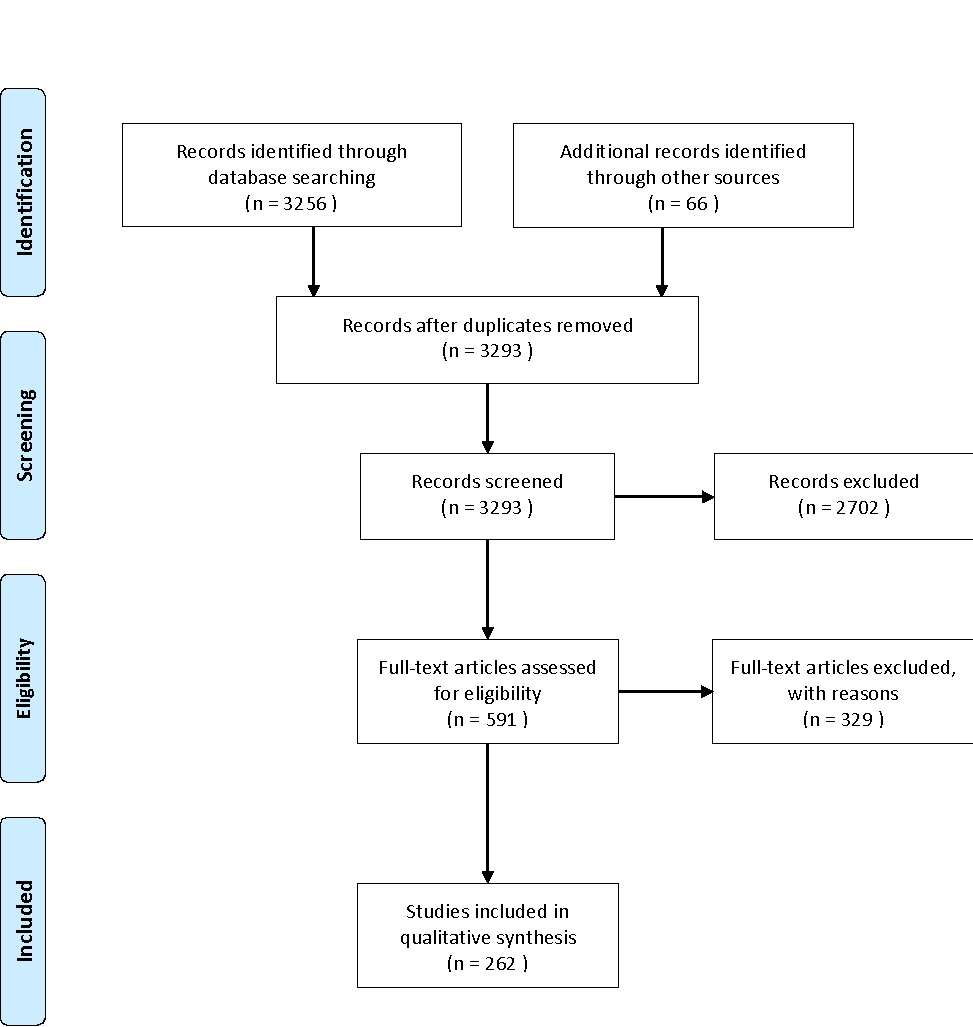


**Fig. S1.** The flowchart illustrating the process of literature search and review, based on PRISMA guidelines, conducted in August and September 2020 (adapted from Moher et al. 2009).

**Scopus and Web of Science search terms used to review the literature for each study species in the study area.**

*Atlantoxerus getulus*

( TITLE-ABS-KEY ( "Atlantoxerus getulus" OR "Barbary ground squirrel" ) AND TITLE-ABS-KEY ( europe* OR "european union" OR EU OR Spain OR introduc* OR invasi* OR establish* OR alien OR invasive OR ias OR allochthonous OR exotic OR "Aichi target 9" OR "EU biodiversity strategy" OR "europe* biodiversity strategy" OR "EU IAS regulation" OR "Europe* IAS regulation" OR “Union List” OR "propagule pressure" OR "colonization pressure" OR "life-history trait*" OR "life history trait*" OR trait* OR "risk assessment*" OR "impact assessment*" OR "environmental impact*" OR "socio-economic impact*" OR "socio economic impact*" OR "economic impact*" ) ) AND ( LIMIT-TO ( SUBJAREA , "AGRI" ) OR LIMIT-TO ( SUBJAREA , "ENVI" ) OR LIMIT-TO ( SUBJAREA , "EART" ) ) AND ( LIMIT-TO ( LANGUAGE , "English" ) )

*Axis axis*

( TITLE-ABS-KEY ( "Axis axis" OR "Indian spotted deer" OR chital* OR “Spotted deer” OR “Axis deer”) AND TITLE-ABS-KEY ( europe* OR "european union" OR EU OR Croatia OR Ukraine OR introduc* OR invasi* OR establish* OR alien OR invasive OR ias OR allochthonous OR exotic OR "Aichi target 9" OR "EU biodiversity strategy" OR "europe* biodiversity strategy" OR "EU IAS regulation" OR "Europe* IAS regulation" OR “Union List” OR "propagule pressure" OR "colonization pressure" OR "life-history trait*" OR "life history trait*" OR trait* OR "risk assessment*" OR "impact assessment*" OR "environmental impact*" OR "socio-economic impact*" OR "socio economic impact*" OR "economic impact*" ) ) AND ( LIMIT-TO ( SUBJAREA , "AGRI" ) OR LIMIT-TO ( SUBJAREA , "ENVI" ) OR LIMIT-TO ( SUBJAREA , "EART" ) ) AND ( LIMIT-TO ( LANGUAGE , "English" ) )

*Callosciurus erythraeus*

( TITLE-ABS-KEY ( "Callosciurus erythraeus" OR "Pallas's squirrel" ) AND TITLE-ABS-KEY ( europe* OR "european union" OR EU OR Belgium OR France OR Germany OR Italy OR Netherlands OR “The Netherlands” OR introduc* OR invasi* OR establish* OR alien OR invasive OR ias OR allochthonous OR exotic OR "Aichi target 9" OR "EU biodiversity strategy" OR "europe* biodiversity strategy" OR "EU IAS regulation" OR "Europe* IAS regulation" OR “Union List” OR "propagule pressure" OR "colonization pressure" OR "life-history trait*" OR "life history trait*" OR trait* OR "risk assessment*" OR "impact assessment*" OR "environmental impact*" OR "socio-economic impact*" OR "socio economic impact*" OR "economic impact*" ) ) AND ( LIMIT-TO ( SUBJAREA , "AGRI" ) OR LIMIT-TO ( SUBJAREA , "ENVI" ) OR LIMIT-TO ( SUBJAREA , "EART" ) ) AND ( LIMIT-TO ( LANGUAGE , "English" ) ) AND ( LIMIT-TO ( PUBYEAR , 2020 ) OR LIMIT-TO ( PUBYEAR , 2019 ) OR LIMIT-TO ( PUBYEAR , 2018 ) OR LIMIT-TO ( PUBYEAR , 2017 ) OR LIMIT-TO ( PUBYEAR , 2016 ) OR LIMIT-TO ( PUBYEAR , 2015 ) OR LIMIT-TO ( PUBYEAR , 2014 ))

*Callosciurus finlaysonii*

( TITLE-ABS-KEY ( "Callosciurus finlaysonii " OR "Variable squirrel" OR “Finlayson’s squirrel”) AND TITLE-ABS-KEY ( europe* OR "european union" OR EU OR Italy OR introduc* OR invasi* OR establish* OR alien OR invasive OR ias OR allochthonous OR exotic OR "Aichi target 9" OR "EU biodiversity strategy" OR "europe* biodiversity strategy" OR "EU IAS regulation" OR "Europe* IAS regulation" OR “Union List” OR "propagule pressure" OR "colonization pressure" OR "life-history trait*" OR "life history trait*" OR trait* OR "risk assessment*" OR "impact assessment*" OR "environmental impact*" OR "socio-economic impact*" OR "socio economic impact*" OR "economic impact*" ) ) AND ( LIMIT-TO ( SUBJAREA , "AGRI" ) OR LIMIT-TO ( SUBJAREA , "ENVI" ) OR LIMIT-TO ( SUBJAREA , "EART" ) ) AND ( LIMIT-TO ( LANGUAGE , "English" ) ) AND ( LIMIT-TO ( PUBYEAR , 2020 ) OR LIMIT-TO ( PUBYEAR , 2019 ) OR LIMIT-TO ( PUBYEAR , 2018 ) )

*Castor canadensis*

[excluding] WEB OF SCIENCE CATEGORIES: ( ANTHROPOLOGY OR MATERIALS SCIENCE MULTIDISCIPLINARY OR MATHEMATICAL COMPUTATIONAL BIOLOGY OR OPHTHALMOLOGY OR ORTHOPEDICS OR LINGUISTICS OR PHYSICS FLUIDS PLASMAS OR THERMODYNAMICS OR MATHEMATICS APPLIED OR COMPUTER SCIENCE ARTIFICIAL INTELLIGENCE OR ENGINEERING ELECTRICAL ELECTRONIC OR COMPUTER SCIENCE SOFTWARE ENGINEERING OR DENTISTRY ORAL SURGERY MEDICINE OR HISTORY OR HUMANITIES MULTIDISCIPLINARY OR MECHANICS OR LANGUAGE LINGUISTICS OR EMERGENCY MEDICINE OR COMPUTER SCIENCE INTERDISCIPLINARY APPLICATIONS OR PHYSICS MATHEMATICAL OR COMPUTER SCIENCE THEORY METHODS OR SURGERY OR ART OR CARDIAC CARDIOVASCULAR SYSTEMS OR HEALTH CARE SCIENCES SERVICES OR HISTORY PHILOSOPHY OF SCIENCE OR MATHEMATICS INTERDISCIPLINARY APPLICATIONS OR COMPUTER SCIENCE INFORMATION SYSTEMS OR EDUCATION EDUCATIONAL RESEARCH OR EDUCATION SCIENTIFIC DISCIPLINES OR ENERGY FUELS OR INSTRUMENTS INSTRUMENTATION OR INTERNATIONAL RELATIONS OR HOSPITALITY LEISURE SPORT TOURISM OR PUBLIC ENVIRONMENTAL OCCUPATIONAL HEALTH ) ( TITLE-ABS-KEY ( "Castor canadensis" OR beaver* OR “American beaver”) AND TITLE-ABS-KEY ( europe* OR "european union" OR EU OR Belgium OR Finland OR France OR Germany OR Luxembourg OR Russia OR “Russian Federation” OR introduc* OR invasi* OR establish* OR alien OR invasive OR ias OR allochthonous OR exotic OR "Aichi target 9" OR "EU biodiversity strategy" OR "europe* biodiversity strategy" OR "EU IAS regulation" OR "Europe* IAS regulation" OR “Union List” OR "propagule pressure" OR "colonization pressure" OR "life-history trait*" OR "life history trait*" OR trait* OR "risk assessment*" OR "impact assessment*" OR "environmental impact*" OR "socio-economic impact*" OR "socio economic impact*" OR "economic impact*" ) ) AND ( LIMIT-TO ( SUBJAREA , "AGRI" ) OR LIMIT-TO ( SUBJAREA , "ENVI" ) OR LIMIT-TO ( SUBJAREA , "EART" ) ) AND ( LIMIT-TO ( LANGUAGE , "English" ) ) AND ( LIMIT-TO ( PUBYEAR , 2020 ) OR LIMIT-TO ( PUBYEAR , 2019 ) OR LIMIT-TO ( PUBYEAR , 2018 ) OR LIMIT-TO ( PUBYEAR , 2017 ) OR LIMIT-TO ( PUBYEAR , 2016 ) OR LIMIT-TO ( PUBYEAR , 2015 ) OR LIMIT-TO ( PUBYEAR , 2014 ) OR LIMIT-TO ( PUBYEAR , 2013 ) OR LIMIT-TO ( PUBYEAR , 2012 ) OR LIMIT-TO ( PUBYEAR , 2011 ) OR LIMIT-TO ( PUBYEAR , 2010 ) )

*Cervus nippon*

( TITLE-ABS-KEY ( "Cervus nippon" OR “Sika deer”) AND TITLE-ABS-KEY ( europe* OR "european union" OR EU OR Austria OR Czechia OR “Czech Republic” OR Denmark OR Finland OR France OR Germany OR Hungary OR Ireland OR Lithuania OR Poland OR Russia OR “Russian Federation” OR Switzerland OR “United Kingdom” OR UK OR Ukraine OR introduc* OR invasi* OR establish* OR alien OR invasive OR ias OR allochthonous OR exotic OR "Aichi target 9" OR "EU biodiversity strategy" OR "europe* biodiversity strategy" OR "EU IAS regulation" OR "Europe* IAS regulation" OR “Union List” OR "propagule pressure" OR "colonization pressure" OR "life-history trait*" OR "life history trait*" OR trait* OR "risk assessment*" OR "impact assessment*" OR "environmental impact*" OR "socio-economic impact*" OR "socio economic impact*" OR "economic impact*" ) ) AND ( LIMIT-TO ( SUBJAREA , "AGRI" ) OR LIMIT-TO ( SUBJAREA , "ENVI" ) OR LIMIT-TO ( SUBJAREA , "EART" ) ) AND ( LIMIT-TO ( LANGUAGE , "English" ) ) AND ( LIMIT-TO ( PUBYEAR , 2020 ) OR LIMIT-TO ( PUBYEAR , 2019 ) OR LIMIT-TO ( PUBYEAR , 2018 ) OR LIMIT-TO ( PUBYEAR , 2017 ) OR LIMIT-TO ( PUBYEAR , 2016 ) OR LIMIT-TO ( PUBYEAR , 2015 ) OR LIMIT-TO ( PUBYEAR , 2014 ) OR LIMIT-TO ( PUBYEAR , 2013 ) OR LIMIT-TO ( PUBYEAR , 2012 ) OR LIMIT-TO ( PUBYEAR , 2011 ) OR LIMIT-TO ( PUBYEAR , 2010 ) OR LIMIT-TO ( PUBYEAR , 2009 ) )

*Eutamias sibiricus*

( TITLE-ABS-KEY ( "Eutamias sibiricus" OR “Tamias sibiricus” OR "Siberian chipmunk" ) AND TITLE-ABS-KEY ( europe* OR "european union" OR EU OR Belgium OR Denmark OR France OR Germany OR Ireland OR Italy OR Netherlands OR “The Netherlands” OR Russia OR “Russian Federation” OR Spain OR Switzerland OR “United Kingdom” OR UK OR introduc* OR invasi* OR establish* OR alien OR invasive OR ias OR allochthonous OR exotic OR "Aichi target 9" OR "EU biodiversity strategy" OR "europe* biodiversity strategy" OR "EU IAS regulation" OR "Europe* IAS regulation" OR “Union List” OR "propagule pressure" OR "colonization pressure" OR "life-history trait*" OR "life history trait*" OR trait* OR "risk assessment*" OR "impact assessment*" OR "environmental impact*" OR "socio-economic impact*" OR "socio economic impact*" OR "economic impact*" ) ) AND ( LIMIT-TO ( SUBJAREA , "AGRI" ) OR LIMIT-TO ( SUBJAREA , "ENVI" ) OR LIMIT-TO ( SUBJAREA , "EART" ) ) AND ( LIMIT-TO ( LANGUAGE , "English" ) )

*Herpestes auropunctatus*

( TITLE-ABS-KEY ("Herpestes javanic*" OR "Herpestes auropunctat*" OR "Urva javanic*" OR "Urva auropunctat*" OR "Small Indian mongoose" ) AND TITLE-ABS-KEY ( europe* OR "european union" OR EU OR Albania OR “Bosnia and Herzegovina” OR “Bosnia-Herzegovina” OR Croatia OR Montenegro OR introduc* OR invasi* OR establish* OR alien OR invasive OR ias OR allochthonous OR exotic OR "Aichi target 9" OR "EU biodiversity strategy" OR "europe* biodiversity strategy" OR "EU IAS regulation" OR "Europe* IAS regulation" OR “Union List” OR "propagule pressure" OR "colonization pressure" OR "life-history trait*" OR "life history trait*" OR trait* OR "risk assessment*" OR "impact assessment*" OR "environmental impact*" OR "socio-economic impact*" OR "socio economic impact*" OR "economic impact*" ) ) AND ( LIMIT-TO ( SUBJAREA , "AGRI" ) OR LIMIT-TO ( SUBJAREA , "ENVI" ) OR LIMIT-TO ( SUBJAREA , "EART" ) ) AND ( LIMIT-TO ( LANGUAGE , "English" ) ) AND ( LIMIT-TO ( PUBYEAR , 2020 ) OR LIMIT-TO ( PUBYEAR , 2019 ) OR LIMIT-TO ( PUBYEAR , 2018 ) OR LIMIT-TO ( PUBYEAR , 2017 ) OR LIMIT-TO ( PUBYEAR , 2016 ) OR LIMIT-TO ( PUBYEAR , 2015 ) )

*Muntiacus reevesi*

( TITLE-ABS-KEY ( "Muntiacus reevesi" OR “Reeves’ muntjac” OR "Reeves muntjac" ) AND TITLE-ABS-KEY ( europe* OR "european union" OR EU OR Belgium OR Denmark OR Ireland OR Netherlands OR “The Netherlands” OR “United Kingdom” OR UK OR introduc* OR invasi* OR establish* OR alien OR invasive OR ias OR allochthonous OR exotic OR "Aichi target 9" OR "EU biodiversity strategy" OR "europe* biodiversity strategy" OR "EU IAS regulation" OR “Union List” OR "Europe* IAS regulation" OR "propagule pressure" OR "colonization pressure" OR "life-history trait*" OR "life history trait*" OR trait* OR "risk assessment*" OR "impact assessment*" OR "environmental impact*" OR "socio-economic impact*" OR "socio economic impact*" OR "economic impact*" ) ) AND ( LIMIT-TO ( SUBJAREA , "AGRI" ) OR LIMIT-TO ( SUBJAREA , "ENVI" ) OR LIMIT-TO ( SUBJAREA , "EART" ) ) AND ( LIMIT-TO ( LANGUAGE , "English" ) )

*Myocastor coypus*

( TITLE-ABS-KEY ( "Myocastor coypus" OR “coypu*” OR “nutria”) AND TITLE-ABS-KEY ( europe* OR "european union" OR EU OR Austria OR Belarus OR Belgium OR Bulgaria OR Croatia OR Czechia OR “Czech Republic” OR Denmark OR France OR Germany OR Greece OR Hungary OR Ireland OR Italy OR Luxembourg OR Macedonia OR Montenegro OR Netherlands OR “The Netherlands” OR Norway OR Poland OR Romania OR Serbia OR Slovakia OR Slovenia OR Spain OR Sweden OR Switzerland OR “United Kingdom” OR UK OR Ukraine OR introduc* OR invasi* OR establish* OR alien OR invasive OR ias OR allochthonous OR exotic OR "Aichi target 9" OR "EU biodiversity strategy" OR "europe* biodiversity strategy" OR "EU IAS regulation" OR "Europe* IAS regulation" OR “Union List” OR "propagule pressure" OR "colonization pressure" OR "life-history trait*" OR "life history trait*" OR trait* OR "risk assessment*" OR "impact assessment*" OR "environmental impact*" OR "socio-economic impact*" OR "socio economic impact*" OR "economic impact*" ) ) AND ( LIMIT-TO ( SUBJAREA , "AGRI" ) OR LIMIT-TO ( SUBJAREA , "ENVI" ) OR LIMIT-TO ( SUBJAREA , "EART" ) ) AND ( LIMIT-TO ( LANGUAGE , "English" ) ) AND ( LIMIT-TO ( PUBYEAR , 2020 ) OR LIMIT-TO ( PUBYEAR , 2019 ) OR LIMIT-TO ( PUBYEAR , 2018 ) OR LIMIT-TO ( PUBYEAR , 2017 ) OR LIMIT-TO ( PUBYEAR , 2016 ) OR LIMIT-TO ( PUBYEAR , 2015 ) OR LIMIT-TO ( PUBYEAR , 2014 ) )

*Nasua nasua*

( TITLE-ABS-KEY ( "Nasua nasua" OR “South American coati” OR “ring-tailed coati” ) AND TITLE-ABS-KEY ( europe* OR "european union" OR EU OR Belgium OR France OR Germany OR Spain OR introduc* OR invasi* OR establish* OR alien OR invasive OR ias OR allochthonous OR exotic OR "Aichi target 9" OR "EU biodiversity strategy" OR "europe* biodiversity strategy" OR "EU IAS regulation" OR "Europe* IAS regulation" OR “Union List” OR "propagule pressure" OR "colonization pressure" OR "life-history trait*" OR "life history trait*" OR trait* OR "risk assessment*" OR "impact assessment*" OR "environmental impact*" OR "socio-economic impact*" OR "socio economic impact*" OR "economic impact*" ) ) AND ( LIMIT-TO ( SUBJAREA , "AGRI" ) OR LIMIT-TO ( SUBJAREA , "ENVI" ) OR LIMIT-TO ( SUBJAREA , "EART" ) ) AND ( LIMIT-TO ( LANGUAGE , "English" ) ) AND ( LIMIT-TO ( PUBYEAR , 2020 ) OR LIMIT-TO ( PUBYEAR , 2019 ) OR LIMIT-TO ( PUBYEAR , 2018 ) OR LIMIT-TO ( PUBYEAR , 2017 ) OR LIMIT-TO ( PUBYEAR , 2016 ) OR LIMIT-TO ( PUBYEAR , 2015 ) )

*Neovison vison*

( TITLE-ABS-KEY ( "Neovison vison" OR “American mink”) AND TITLE-ABS-KEY ( europe* OR "european union" OR EU OR Albania OR Andorra OR Austria OR Belarus OR Belgium OR Czechia OR “Czech Republic” OR Denmark OR Estonia OR Finland OR France OR Germany OR Greece OR Hungary OR Iceland OR Ireland OR Italy OR Latvia OR Lithuania OR Luxembourg OR Macedonia OR “North Macedonia” OR Montenegro OR Netherlands OR “The Netherlands” OR Norway OR Poland OR Portugal OR Romania OR Russia OR “Russian federation” OR Slovakia OR Slovenia OR Serbia OR Spain OR Sweden OR Switzerland OR “United Kingdom” OR UK OR Ukraine OR introduc* OR invasi* OR establish* OR alien OR invasive OR ias OR allochthonous OR exotic OR "Aichi target 9" OR "EU biodiversity strategy" OR "europe* biodiversity strategy" OR "EU IAS regulation" OR "Europe* IAS regulation" OR “Union List” OR "propagule pressure" OR "colonization pressure" OR "life-history trait*" OR "life history trait*" OR trait* OR "risk assessment*" OR "impact assessment*" OR "environmental impact*" OR "socio-economic impact*" OR "socio economic impact*" OR "economic impact*" ) ) AND ( LIMIT-TO ( SUBJAREA , "AGRI" ) OR LIMIT-TO ( SUBJAREA , "ENVI" ) OR LIMIT-TO ( SUBJAREA , "EART" ) ) AND ( LIMIT-TO ( LANGUAGE , "English" ) ) AND ( LIMIT-TO ( PUBYEAR , 2020 ) OR LIMIT-TO ( PUBYEAR , 2019 ) OR LIMIT-TO ( PUBYEAR , 2018 ) OR LIMIT-TO ( PUBYEAR , 2017 ) OR LIMIT-TO ( PUBYEAR , 2016 ) )

*Nyctereutes procyonoides*

( TITLE-ABS-KEY ( "Nyctereutes procyonoides" OR “Racoon dog*” ) AND TITLE-ABS-KEY ( europe* OR "european union" OR EU OR Albania OR Austria OR Belarus OR Belgium OR “Bosnia and Herzegovina” OR “Bosnia-Herzegovina” OR Bulgaria OR Croatia OR Czechia OR “Czech Republic” OR Denmark OR Estonia OR Finland OR France OR Germany OR Greece OR Hungary OR Italy OR Latvia OR Liechtenstein OR Lithuania OR Luxembourg OR Macedonia OR “North Macedonia” OR Moldova OR Montenegro OR Netherlands OR “The Netherlands” OR Norway OR Poland OR Romania OR Russia OR “Russian Federation” OR Serbia OR Slovakia OR Slovenia OR Sweden OR Switzerland OR Ukraine OR introduc* OR invasi* OR establish* OR alien OR invasive OR ias OR allochthonous OR exotic OR "Aichi target 9" OR "EU biodiversity strategy" OR "europe* biodiversity strategy" OR "EU IAS regulation" OR "Europe* IAS regulation" OR “Union List” OR "propagule pressure" OR "colonization pressure" OR "life-history trait*" OR "life history trait*" OR trait* OR "risk assessment*" OR "impact assessment*" OR "environmental impact*" OR "socio-economic impact*" OR "socio economic impact*" OR "economic impact*" ) ) AND ( LIMIT-TO ( SUBJAREA , "AGRI" ) OR LIMIT-TO ( SUBJAREA , "ENVI" ) OR LIMIT-TO ( SUBJAREA , "EART" ) ) AND ( LIMIT-TO ( LANGUAGE , "English" ) ) AND ( LIMIT-TO ( PUBYEAR , 2020 ) OR LIMIT-TO ( PUBYEAR , 2019 ) OR LIMIT-TO ( PUBYEAR , 2018 ) OR LIMIT-TO ( PUBYEAR , 2017 ) OR LIMIT-TO ( PUBYEAR , 2016 ) OR LIMIT-TO ( PUBYEAR , 2015 ) )

*Ondatra zibethicus*

( TITLE-ABS-KEY ( "Ondatra zibethicus" OR muskrat* ) AND TITLE-ABS-KEY ( europe* OR "european union" OR EU OR Andorra OR Austria OR Belarus OR Belgium OR “Bosnia and Herzegovina” OR “Bosnia-Herzegovina” OR Bulgaria OR Croatia OR Czechia OR “Czech Republic” OR Denmark OR Estonia OR Finland OR France OR Germany OR Greece OR Hungary OR Ireland OR Italy OR Latvia OR Liechtenstein OR Lithuania OR Luxembourg OR Moldova OR Montenegro OR Netherlands OR “The Netherlands” OR Norway OR Poland OR Romania OR Russia OR “Russian Federation” OR Serbia OR Slovakia OR Slovenia OR Spain OR Sweden OR Switzerland OR “United Kingdom” OR UK OR Ukraine OR introduc* OR invasi* OR establish* OR alien OR invasive OR ias OR allochthonous OR exotic OR "Aichi target 9" OR "EU biodiversity strategy" OR "europe* biodiversity strategy" OR "EU IAS regulation" OR "Europe* IAS regulation" OR “Union List” OR "propagule pressure" OR "colonization pressure" OR "life-history trait*" OR "life history trait*" OR trait* OR "risk assessment*" OR "impact assessment*" OR "environmental impact*" OR "socio-economic impact*" OR "socio economic impact*" OR "economic impact*" ) ) AND ( LIMIT-TO ( SUBJAREA , "AGRI" ) OR LIMIT-TO ( SUBJAREA , "ENVI" ) OR LIMIT-TO ( SUBJAREA , "EART" ) ) AND ( LIMIT-TO ( LANGUAGE , "English" ) ) AND ( LIMIT-TO ( PUBYEAR , 2020 ) OR LIMIT-TO ( PUBYEAR , 2019 ) OR LIMIT-TO ( PUBYEAR , 2018 ) OR LIMIT-TO ( PUBYEAR , 2017 ) OR LIMIT-TO ( PUBYEAR , 2016 ) OR LIMIT-TO ( PUBYEAR , 2015 ) )

*Procyon lotor*

( TITLE-ABS-KEY ( "Procyon lotor" OR raccoon* OR “Northern raccoon” AND NOT “raccoon dog”) AND TITLE-ABS-KEY ( europe* OR "european union" OR EU OR Austria OR Belarus OR Belgium OR Croatia OR Czechia OR “Czech Republic” OR Denmark OR Estonia OR France OR Germany OR Hungary OR Ireland OR Italy OR Liechtenstein OR Lithuania OR Luxembourg OR Netherlands OR “The Netherlands” OR Poland OR Romania OR Russia OR “Russian Federation” OR Serbia OR Slovakia OR Slovenia OR Spain OR Switzerland OR Ukraine OR introduc* OR invasi* OR establish* OR alien OR invasive OR ias OR allochthonous OR exotic OR "Aichi target 9" OR "EU biodiversity strategy" OR "europe* biodiversity strategy" OR "EU IAS regulation" OR "Europe* IAS regulation" OR “Union List” OR "propagule pressure" OR "colonization pressure" OR "life-history trait*" OR "life history trait*" OR trait* OR "risk assessment*" OR "impact assessment*" OR "environmental impact*" OR "socio-economic impact*" OR "socio economic impact*" OR "economic impact*" ) ) AND ( LIMIT-TO ( SUBJAREA , "AGRI" ) OR LIMIT-TO ( SUBJAREA , "ENVI" ) OR LIMIT-TO ( SUBJAREA , "EART" ) ) AND ( LIMIT-TO ( LANGUAGE , "English" ) ) AND ( LIMIT-TO ( SUBJAREA , "AGRI" ) OR LIMIT-TO ( SUBJAREA , "ENVI" ) OR LIMIT-TO ( SUBJAREA , "EART" ) ) AND ( LIMIT-TO ( LANGUAGE , "English" ) ) AND ( LIMIT-TO ( PUBYEAR , 2020 ) OR LIMIT-TO ( PUBYEAR , 2019 ) OR LIMIT-TO ( PUBYEAR , 2018 ) OR LIMIT-TO ( PUBYEAR , 2017 ) OR LIMIT-TO ( PUBYEAR , 2016 ) OR LIMIT-TO ( PUBYEAR , 2015 ) OR LIMIT-TO ( PUBYEAR , 2014 ) OR LIMIT-TO ( PUBYEAR , 2013 ) OR LIMIT-TO ( PUBYEAR , 2012 ) OR LIMIT-TO ( PUBYEAR , 2011 ) )

*Sciurus carolinensis*

( TITLE-ABS-KEY ( "*Sciurus carolinensis*" OR “Eastern gr*y squirrel” OR “American gr*y squirrel” OR “gr*y squirrel”) AND TITLE-ABS-KEY ( europe* OR "european union" OR EU OR Belgium OR Germany OR Ireland OR Italy OR Netherlands OR “The Netherlands” OR “United Kingdom” OR UK OR introduc* OR invasi* OR establish* OR alien OR invasive OR ias OR allochthonous OR exotic OR "Aichi target 9" OR "EU biodiversity strategy" OR "europe* biodiversity strategy" OR "EU IAS regulation" OR "Europe* IAS regulation" OR “Union List” OR "propagule pressure" OR "colonization pressure" OR "life-history trait*" OR "life history trait*" OR trait* OR "risk assessment*" OR "impact assessment*" OR "environmental impact*" OR "socio-economic impact*" OR "socio economic impact*" OR "economic impact*" ) ) AND ( LIMIT-TO ( SUBJAREA , "AGRI" ) OR LIMIT-TO ( SUBJAREA , "ENVI" ) OR LIMIT-TO ( SUBJAREA , "EART" ) ) AND ( LIMIT-TO ( LANGUAGE , "English" ) ) AND ( LIMIT-TO ( PUBYEAR , 2020 ) OR LIMIT-TO ( PUBYEAR , 2019 ) OR LIMIT-TO ( PUBYEAR , 2018 ) OR LIMIT-TO ( PUBYEAR , 2017 ) OR LIMIT-TO ( PUBYEAR , 2016 ) OR LIMIT-TO ( PUBYEAR , 2015 ) )
