# Supplementary material for: Introduction, spread, and impacts of invasive alien mammal species in Europe
Source: Mamm Rev. 2021 Nov 23;52(2):252–66. doi: 10.1111/mam.12277 (PMC9299096; doi:10.1111/mam.12277)
Supplement: Supplementary file 3 — Appendix S3. Figures illustrating the trends in the published literature, species’ taxonomy, traits, native zoogeographic realms, and pathogen classification. [file MAM-52-252-s001.docx]

**Appendix S3.** Figures illustrating the trends in the published literature, species’ taxonomy, traits, native zoogeographic realms, and pathogens classification.


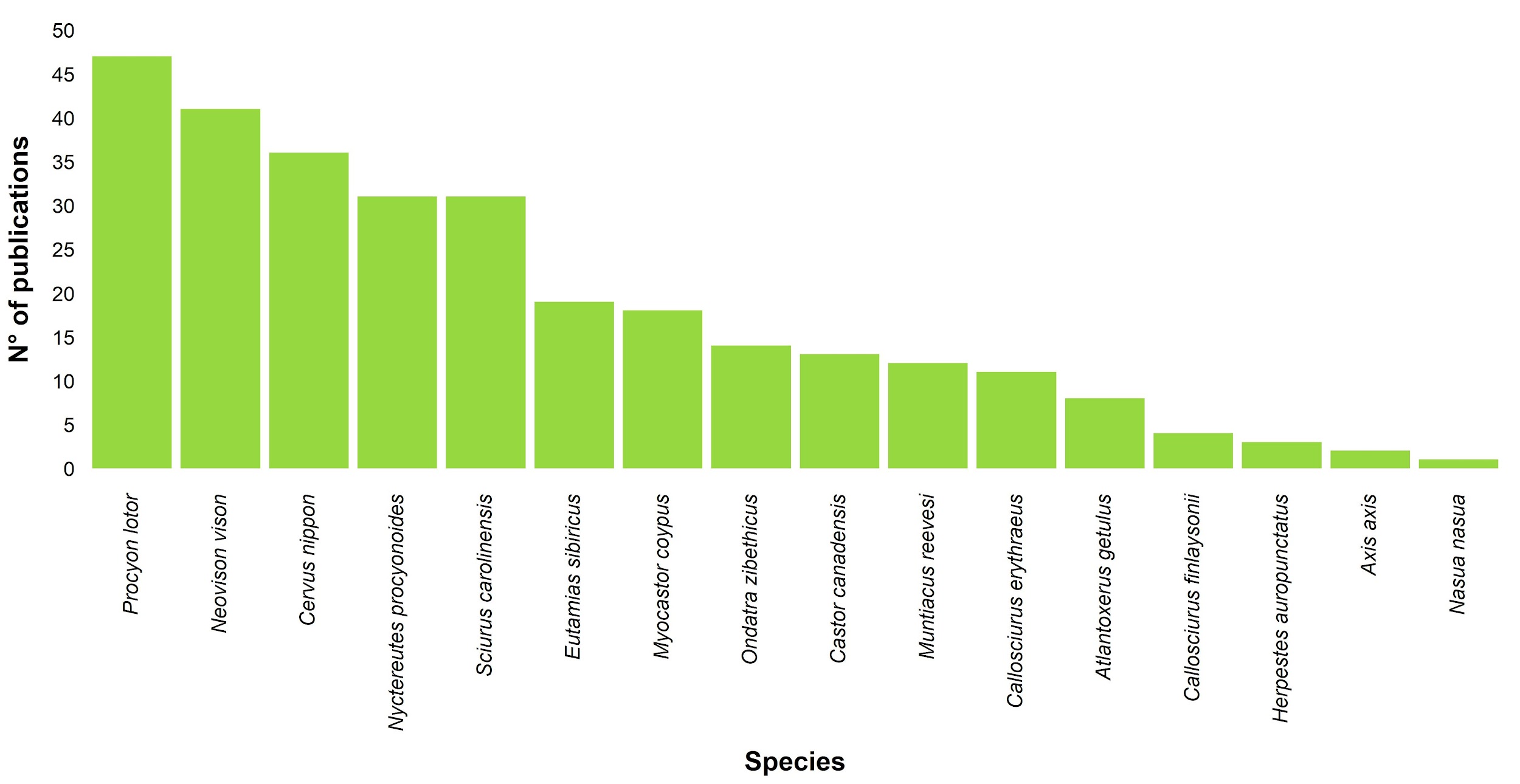


**Fig. S2.** The number of publications resulting from the literature search process collected for each study species in Europe (*n* = 291).


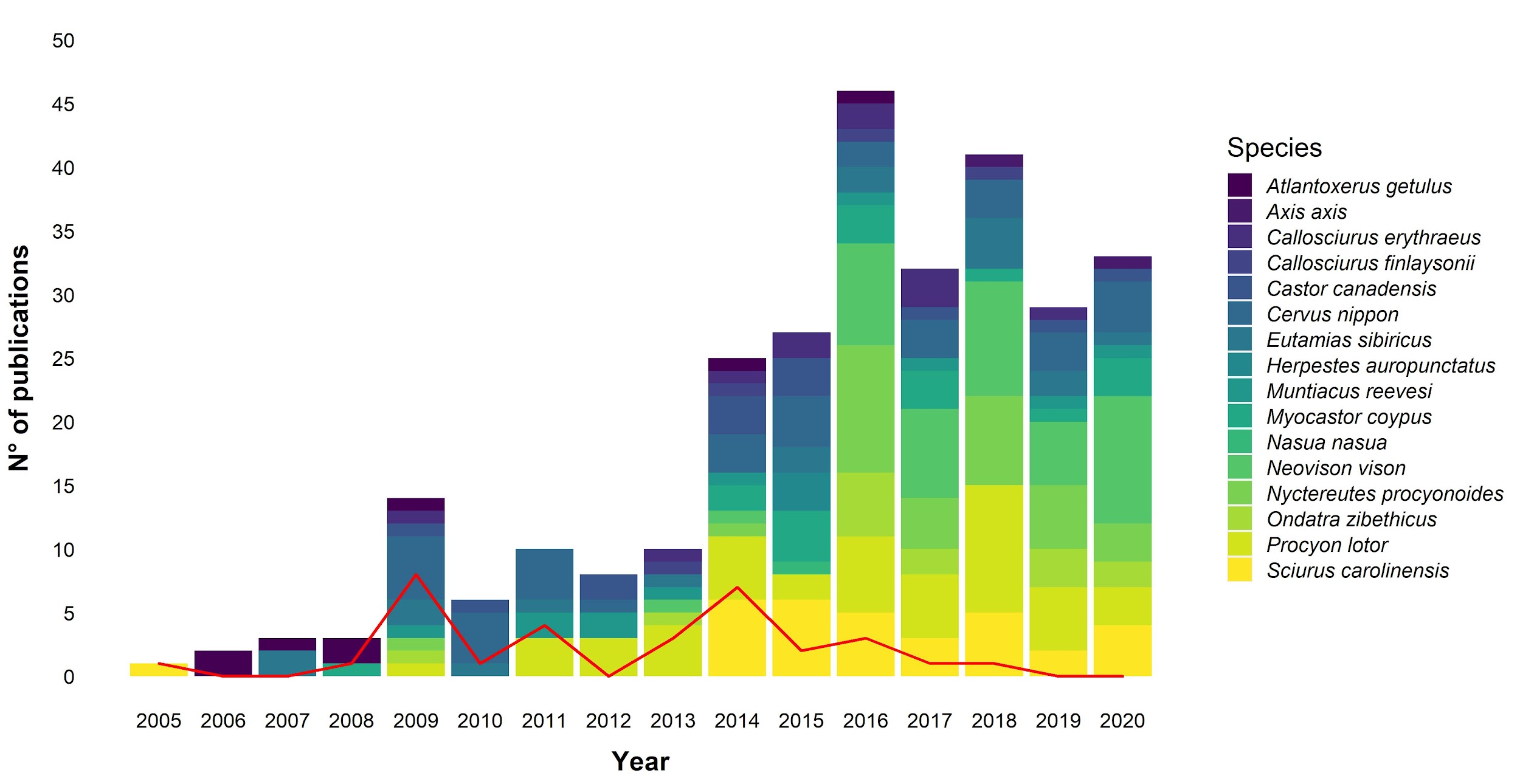


**Fig. S3.** The number of published studies per year for each study species (with duplicates) from 2005 to 2020 (*n* = 290; one publication has no date). The line shows the overall temporal trend of the published datasheets.


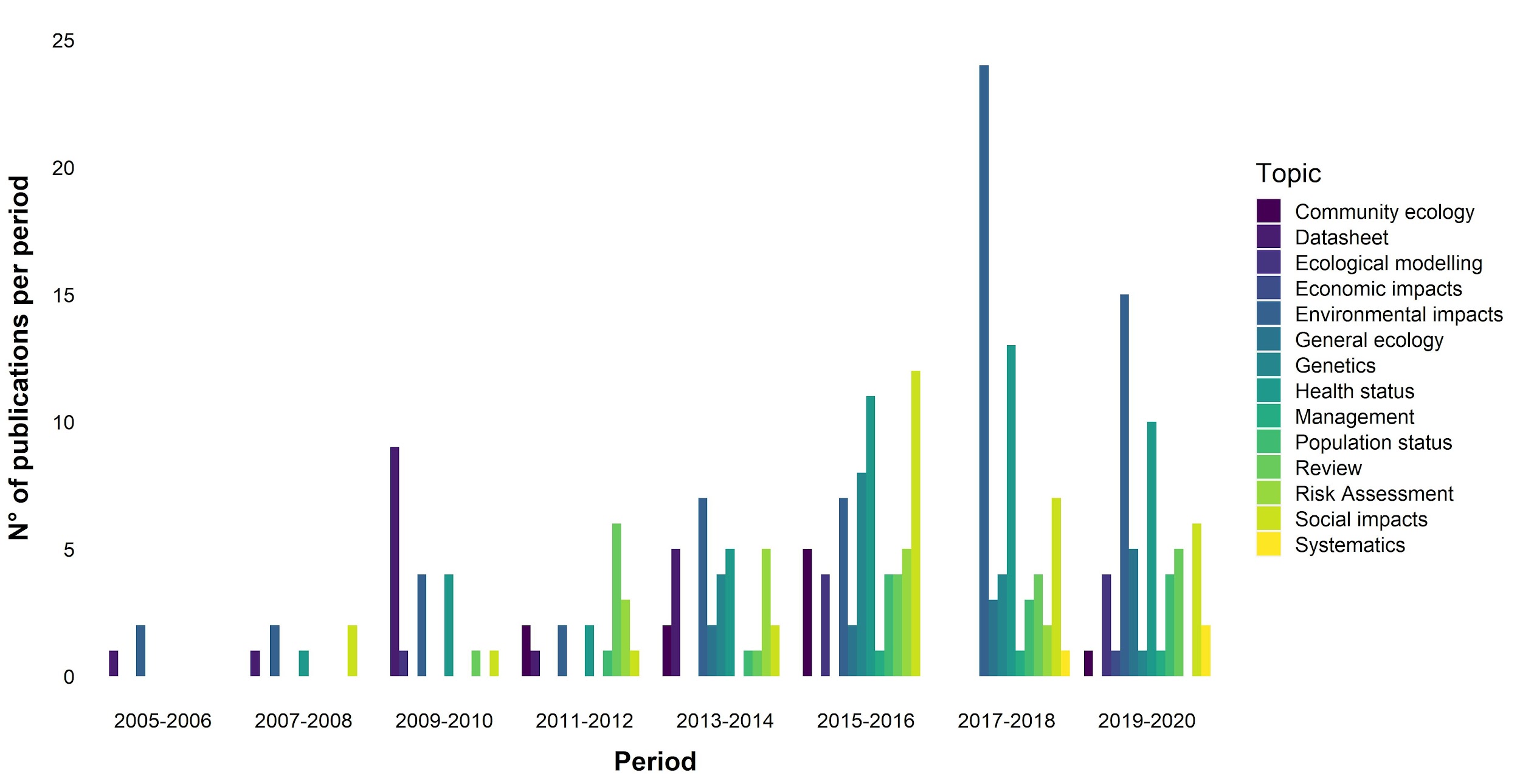


**Fig. S4.** The number of publications for each topic over a two-year period from 2005 to 2020 (*n* = 261; one publication has no date).


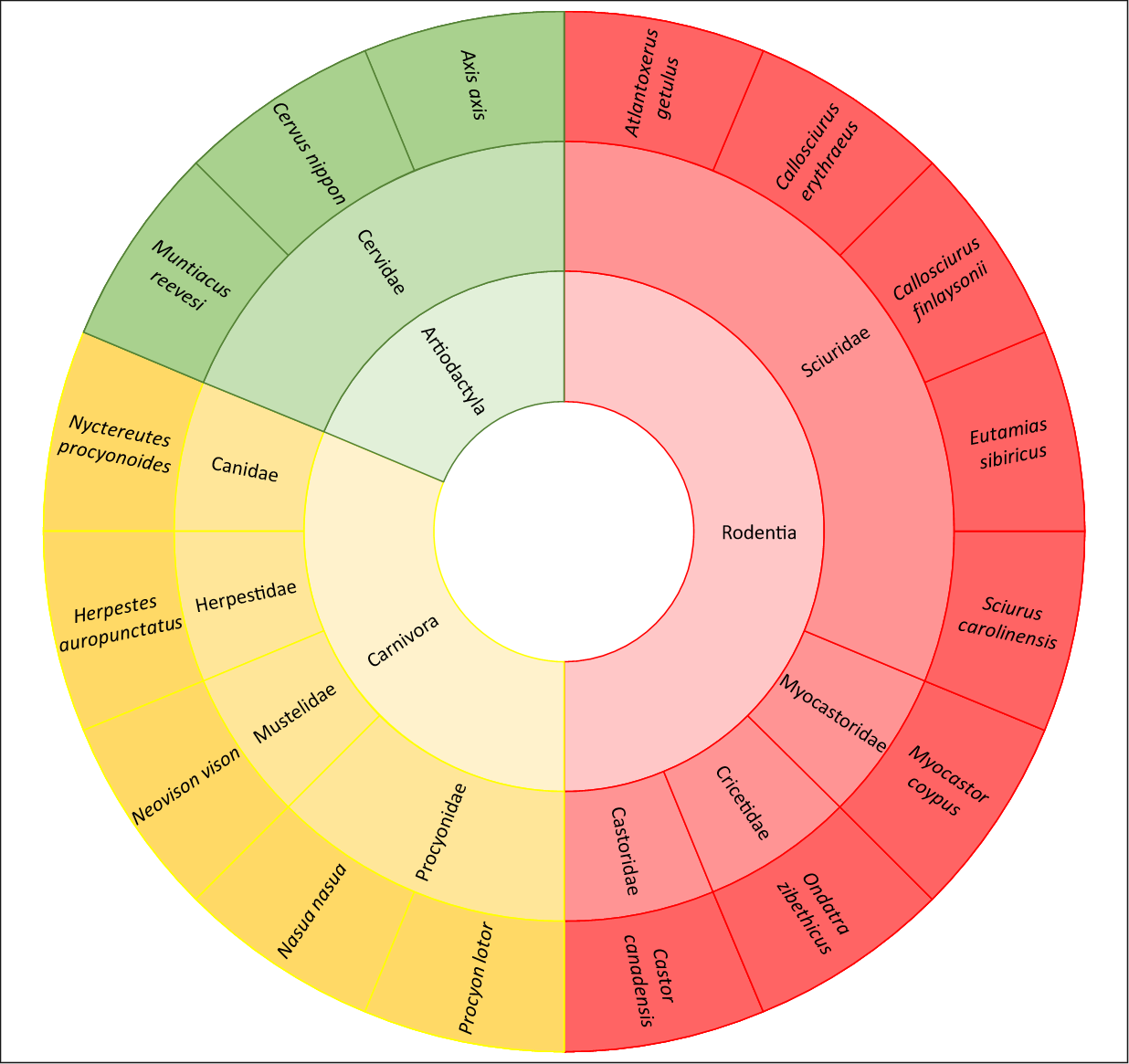


**Fig. S5.** Taxonomic assignment of the study species (*n* = 16). The inner circle represents the orders, the middle circle the families and the outer circle the species.


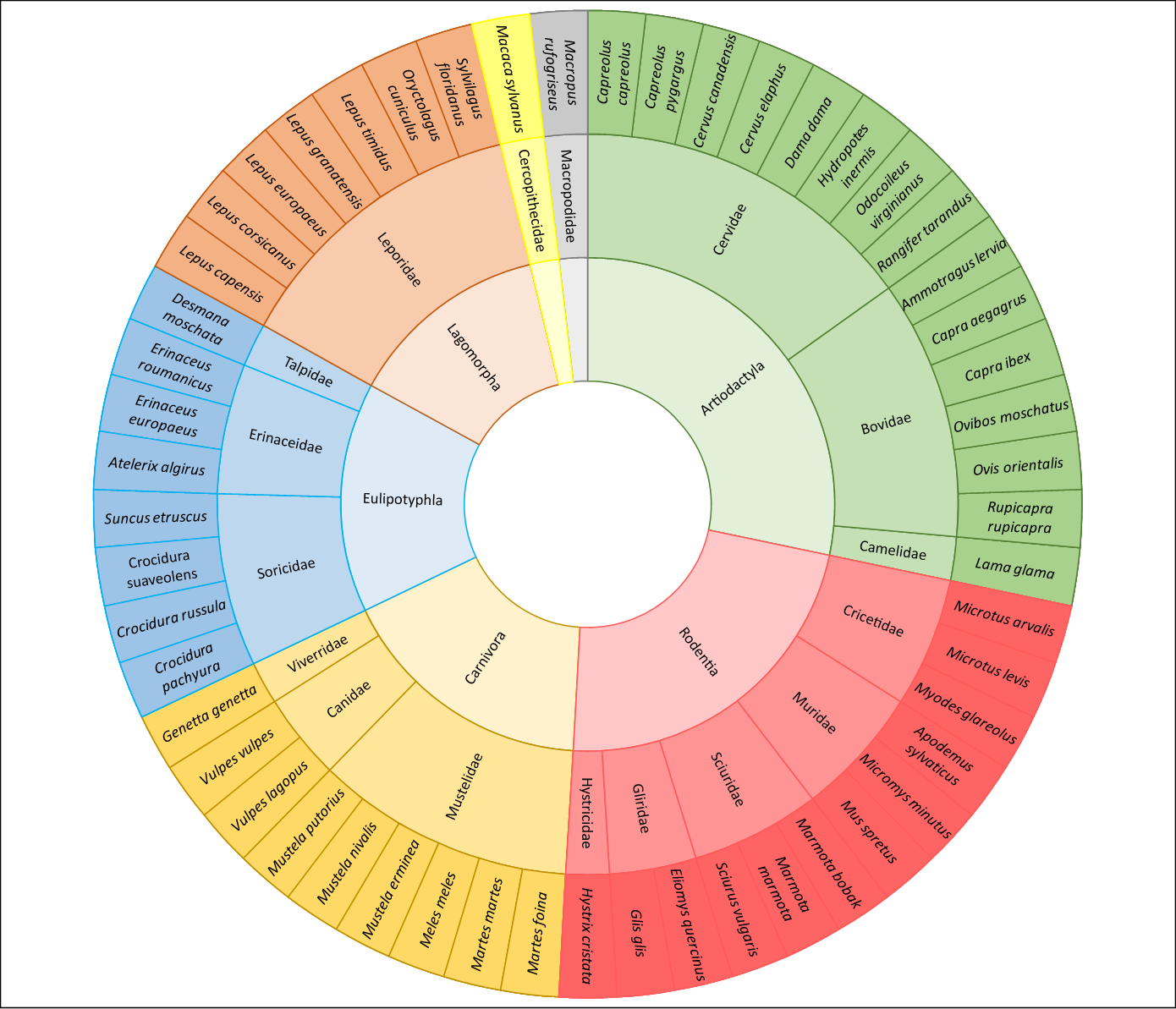


**Fig. S6.** Taxonomic assignment of all the introduced mammals established in Europe (*n* = 53). The inner circle represents the orders, the middle circle the families and the outer circle the species.


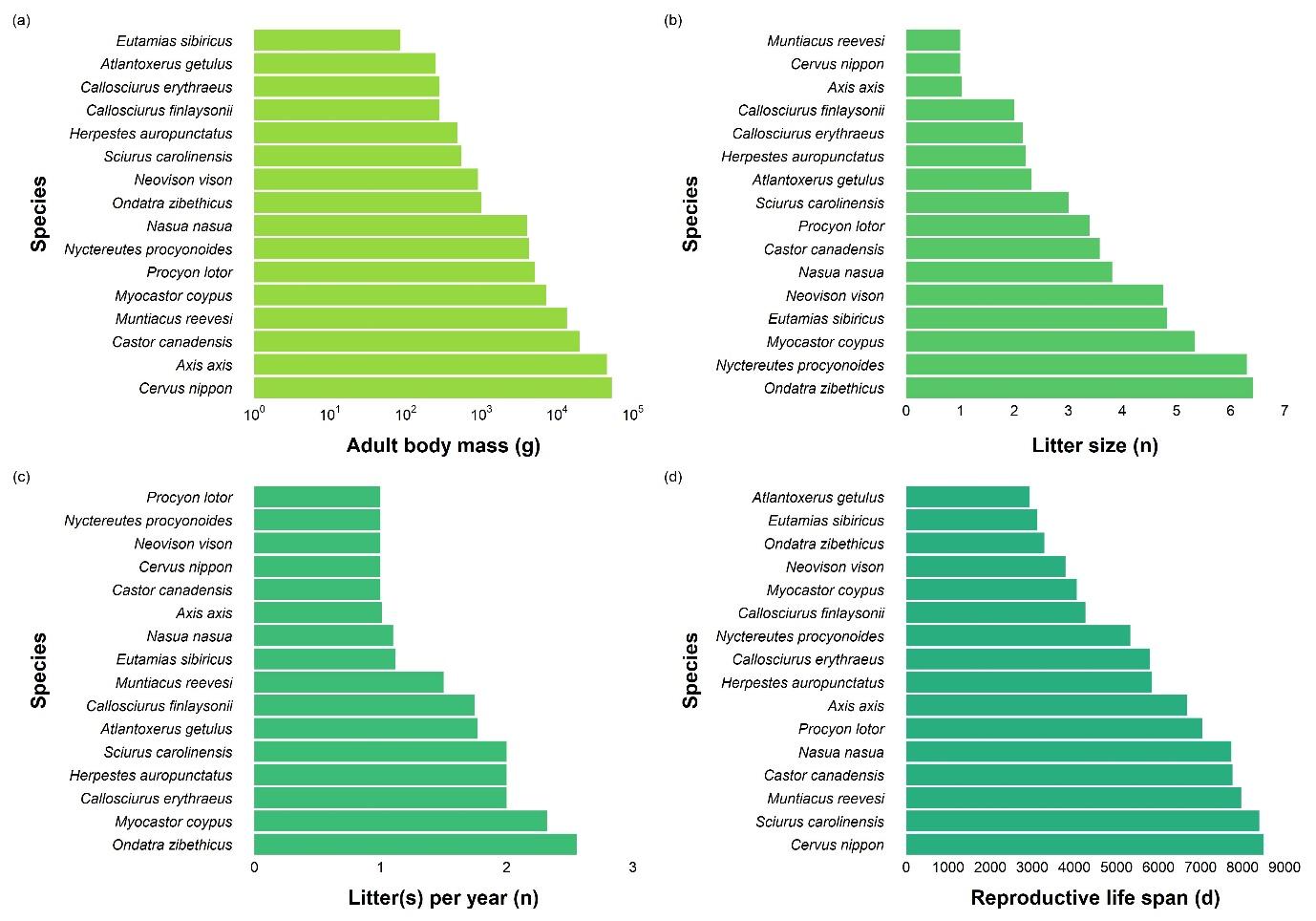


**Fig. S7.** Traits favouring the introduction, establishment, and spread (Capellini et al. 2015; Blackburn et al. 2017) of the study species: (a) adult body mass (log scale, in grams), (b) litter size, (c) litter(s) per year, and (d) generation length (in days).


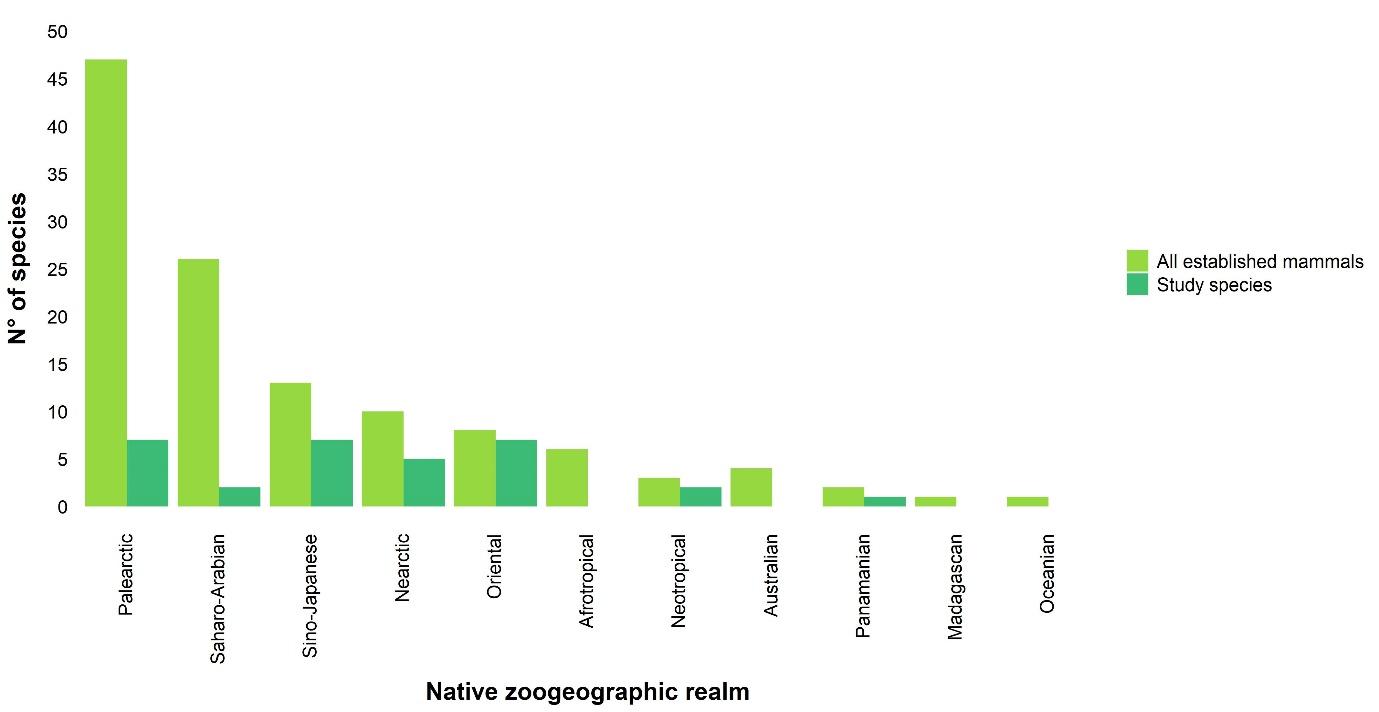


**Fig. S8.** Native zoogeographic realms (Holt et al. 2013) of all the introduced mammals established in Europe (*n* = 119) and of the study species (*n* = 31).

**
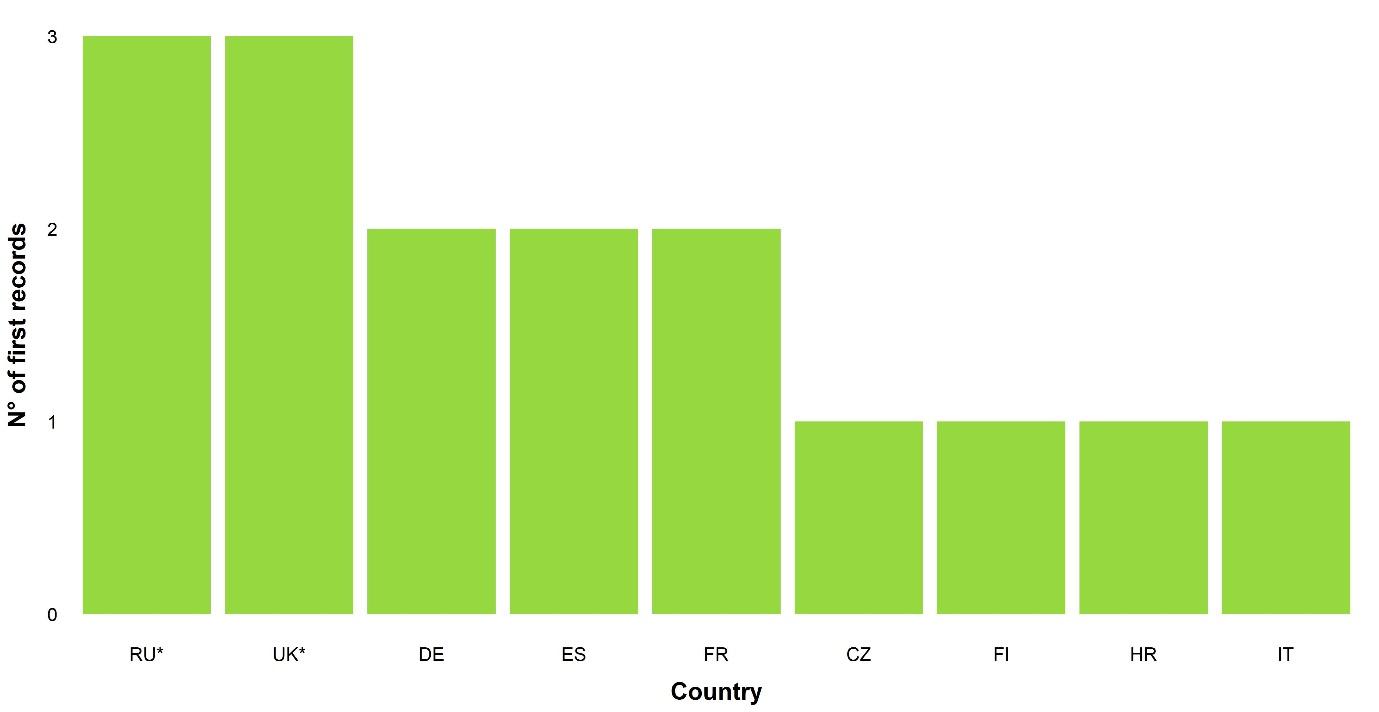
Fig. S9.** First continental records in the countries of Europe (*n* = 16). Countries without invasive mammal species are not shown. Countries marked with an asterisk (*) are not Member States of the EU.


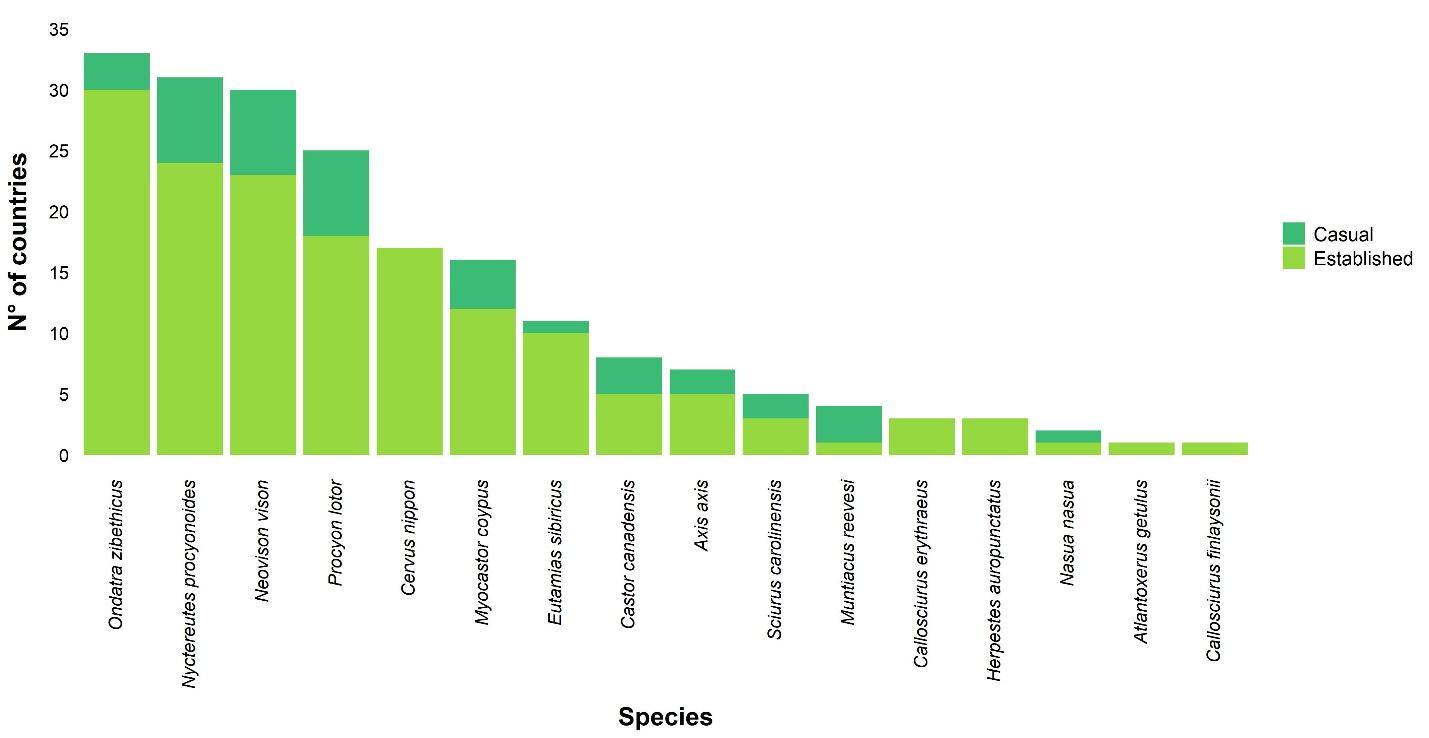


**Fig. S10.** Number of countries in Europe with established and casual presences (*n* = 197) of the study species.


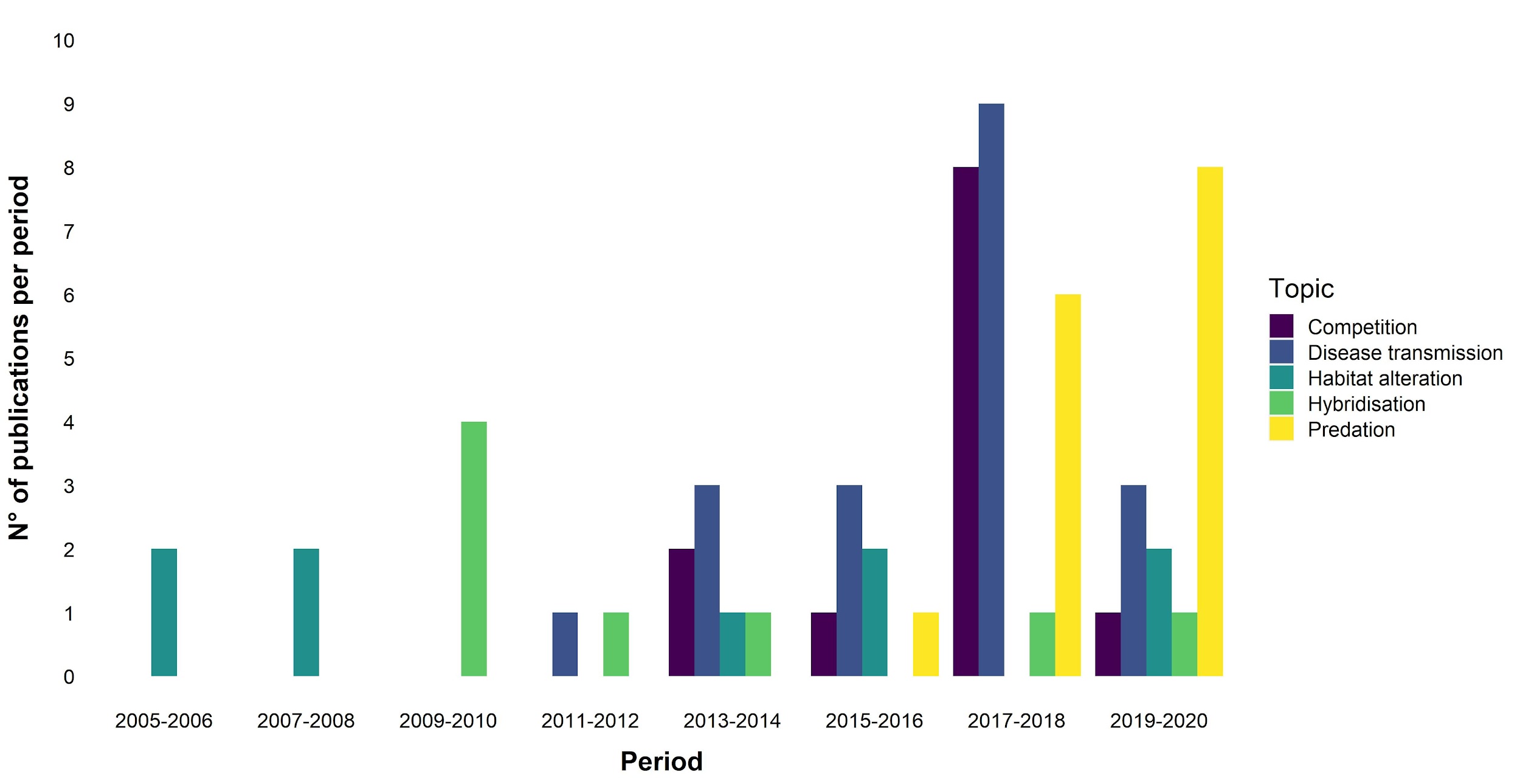


**Fig. S11.** The number of published papers (*n* = 63) regarding environmental impacts of invasive mammal species in Europe, divided per impact categories (following Blackburn et al. 2014).


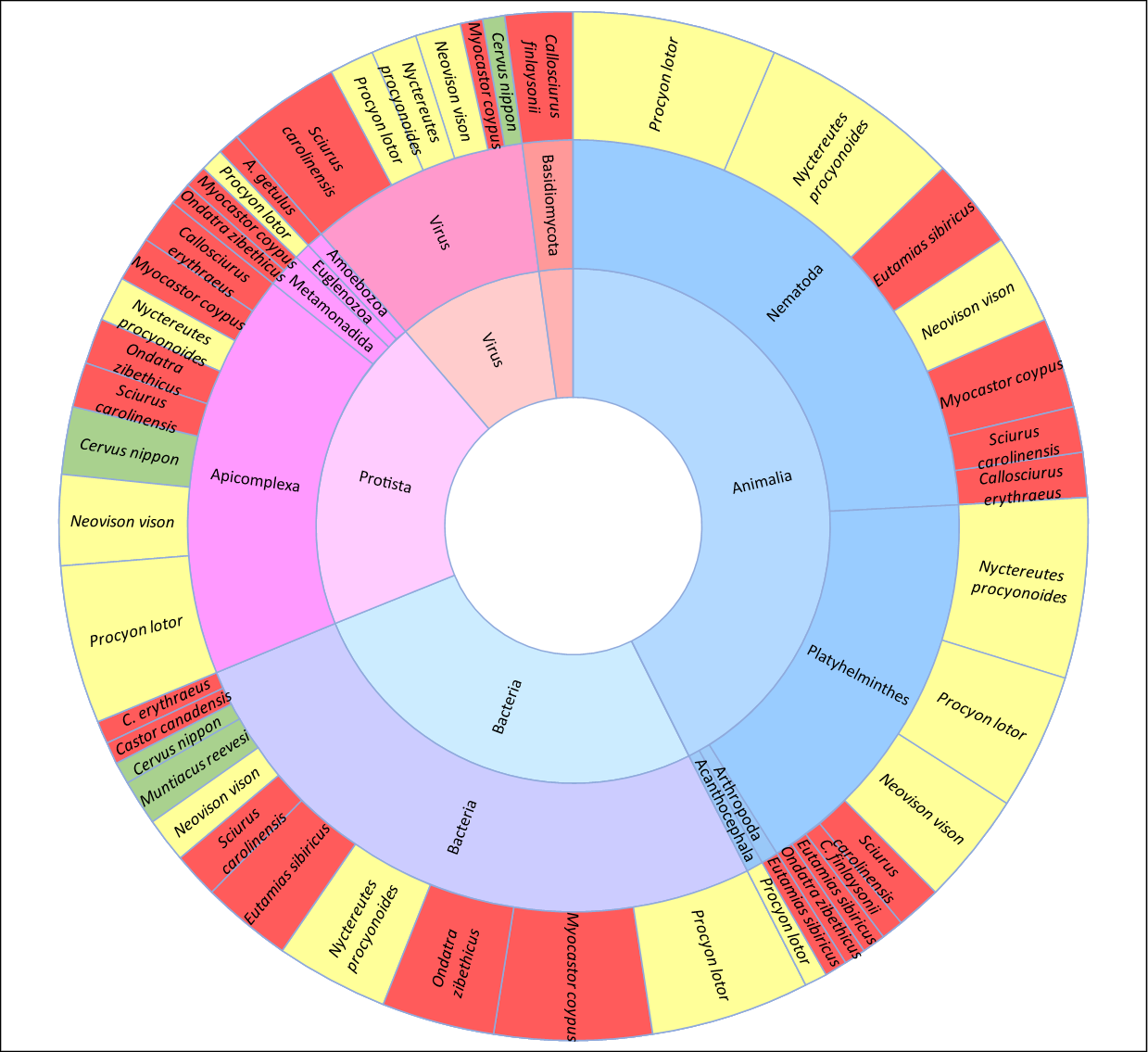


**Fig. S12.** Taxonomic assignments of the pathogens (*n* = 141) and study species (*n* = 13) known to be infected by them. The inner circle represents the Domain or the Kingdom, the middle circle the phyla, and the outer circle the species know to be infected. Species pertaining to the same order are indicated with the same colour (yellow for Carnivora, red for Rodentia, green for Artiodactyla).
