# Supplementary material for: Introduction, spread, and impacts of invasive alien mammal species in Europe
Source: Mamm Rev. 2021 Nov 23;52(2):252–66. doi: 10.1111/mam.12277 (PMC9299096; doi:10.1111/mam.12277)
Supplement: Supplementary file 5 — Appendix S5. List of pathogens known to have been recorded to infect the study species in Europe and list of additional references. [file MAM-52-252-s005.docx]

**Appendix S5.** List of pathogens known to have been recorded to infect the study species in Europe and list of additional references.

| Species | Pathogen | Zoonotic | Country | Prevalence | Reference | Notes |
| --- | --- | --- | --- | --- | --- | --- |
| *Atlantoxerus getulus* | *Acanthamoeba* spp. | YES | ES | 23.50% | Lorenzo-Morales et al. 2007 |  |
| *Callosciurus erythraeus* | Capillariinae | YES | IT | 1% | Mazzamuto et al. 2016 |  |
|  | *Ceratophyllus s. sciurorum* | NO | IT | 50% | Mazzamuto et al. 2016 |  |
|  | *Cryptosporidium* spp. | YES | IT | 2.80% | Prediger et al. 2017 |  |
|  | *Ctenophtalmus agyrtes sardiniensis* | NO | IT | 1% | Mazzamuto et al. 2016 |  |
|  | *Ctenophtalmus* sp. | NO | IT | 1% | Mazzamuto et al. 2016 |  |
|  | *Eimeria* spp. | YES | IT | 4.10% | Hofmannová et al. 2016 |  |
|  | *Ixodes ricinus* |  | IT | 47% | Mazzamuto et al. 2016 |  |
|  | *Mycobacterium leprae* | YES | IT | 0% | Schilling et al. 2019 |  |
|  | *Mycobacterium leprae* | YES | FR | 0% | Schilling et al. 2019 |  |
|  | Spiruridae | NO | IT | 1% | Mazzamuto et al. 2016 |  |
|  | *Strongyloides callosciureus* | NO | IT | 1% | Mazzamuto et al. 2016 |  |
|  | *Strongyloides* sp. | YES | IT | 1% | Mazzamuto et al. 2016 |  |
|  | *Trichuris muris* | NO | IT | 4% | Mazzamuto et al. 2016 |  |
|  | Trombiculidae | NO | IT | 7% | Mazzamuto et al. 2016 |  |
|  | *Trypanoxyuris sciuri* | NO | IT | 5% | Mazzamuto et al. 2016 |  |
| *Callosciurus finlaysonii* | *Cryptococcus neoformans* | YES | IT | 5.60% | Iatta et al. 2015 |  |
|  | *Debaryomyces hansenii* | YES | IT | 0.80% | Iatta et al. 2015 |  |
|  | *Dicrocoelium dendriticum* | YES | IT | 33.30% | d'Ovidio et al*.* 2014 |  |
|  | *Hanseniaspora thailandica* | NO | IT | 3.20% | Iatta et al. 2015 |  |
| Species | Pathogen | Zoonotic | Country | Prevalence | Reference | Notes |
| *Callosciurus finlaysonii* | *Meyerozyma guilliermondii* | YES | IT | 0.80% | Iatta et al. 2015 |  |
| *Castor canadensis* | *Francisella tularensis* | YES | SE |  | Sissonen et al. 2015 | Samples analysed were already infected. |
| *Cervus nippon* | *Anaplasma phagocytophilum* | YES | UK | 50% | Robinson et al. 2009 |  |
|  | *Ashworthius sidemi* | NO | RU |  | Panova et al. 2017 | Probably introduced in Europe with *C. nippon.* |
|  | *Babesia* spp. | YES | CZ | 21.90% | Hrazdilová et al. 2020 |  |
|  | *BlueTongue Virus* (BTV) | NO | IE | 0% | Graham et al. 2017 | Pooled prevalence: sika + fallow + red deer. |
|  | *Border Disease Virus* (BDV) | NO | CZ | 0% | Sedlak et al. 2009 |  |
|  | *Bovine HerpesVirus-1* (BoHV-1) | NO | IE | 1.80% | Graham et al. 2017 | Pooled prevalence: sika + fallow + red deer. |
|  | *Bovine Viral Diarrhoea Virus* (BVDV) | NO | CZ | 0% | Sedlak et al. 2009 |  |
|  | *Bovine Viral Diarrhoea Virus* (BVDV) | NO | IE | 1.50% | Graham et al. 2017 | Pooled prevalence: sika + fallow + red deer. |
|  | *Hepatitis E Virus* (HEV) | YES | DE | 0% | Trojnar et al. 2020 |  |
|  | *Hepatitis E Virus* (HEV) | YES | PL | 0% | Larska et al. 2015 |  |
|  | *Hepatitis E Virus* (HEV) | YES | CZ | 0% | Kubankova et al. 2015 |  |
|  | *Lipoptena fortisetosa* | NO | EE |  | Mihalca et al. 2019 | Probably introduced in Europe with *C. nippon.* |
|  | *Onchocerca flexuosa* | NO | CZ | 16.70% | Dykova & Blazek, 1972 | Can be a host. |
|  | *Sarcocystis* spp. | YES | LT | 100% | Prakas et al. 2016 | Farm bred animals. |
|  | *Sarcocystis* spp. | YES | LT | 92% | Rudaitytė-Lukošienė et al. 2018 |  |
|  | *Schmallenberg Virus* (SBV) | NO | IE | 9.70% | Graham et al. 2017 | Pooled prevalence: sika + fallow + red deer. |
|  | *Toxoplasma gondii* | YES | CZ | 50% | Lorencova et al. 2015 | Antibodies. DNA prevalence: 0%. |
|  | *Trichuris discolor* | NO | CZ | 5.20% | Nechybová et al. 2018 |  |
|  | *Trichuris ovis* | NO | CZ | 1.70% | Nechybová et al. 2018 |  |
|  | *Wehrdickmansia cervipedis* | NO | CZ | 16.70% | Dykova & Blazek, 1972 |  |
| *Eutamias sibiricus* | *Aonchotheca annulosa* | NO | FR | 47% | Pisanu et al. 2007 |  |
| Species | Pathogen | Zoonotic | Country | Prevalence | Reference | Notes |
| *Eutamias sibiricus* | *Aonchotheca annulosa* | NO | FR | 40.50% | Pisanu et al. 2009 |  |
|  | Ascaroidea | YES | FR | 2.40% | Pisanu et al. 2009 |  |
|  | *Borrelia burgdoferi sensu lato* | YES | FR | 33.30% | Vourc’h et al. 2007 |  |
|  | *Borrelia burgdoferi sensu lato* | YES | FR | 35% | Marsot et al. 2011 |  |
|  | *Borrelia burgdoferi sensu lato* | YES | FR | 5%-60% | Marsot et al. 2013 |  |
|  | *Borrelia lusitaniae* | YES | IT |  | Mori et al. 2018b | *B. lusitaniae* and *R. monacensis* were present in ticks of the chipmunks. |
|  | *Brevistriata skrjabini* |  | FR | 90.5% | Pisanu et al. 2009 |  |
|  | *Brevistriata skrjabini* |  | FR | 87% | Pisanu et al. 2007 |  |
|  | *Hymenolepis* spp. | YES | IT | 0% | d’Ovidio et al. 2015 |  |
|  | *Mycobacterium leprae* | YES | FR | 0% | Schilling et al. 2019 |  |
|  | Oxyuridea | YES | FR | 2.40% | Pisanu et al. 2009 |  |
|  | *Rickettsia monacensis* | YES | IT |  | Mori et al. 2018b | *B. lusitaniae* and *R. monacensis* were present in ticks of the chipmunks. |
|  | *Strongyloides callosciureus* | NO | FR | 19% | Pisanu et al. 2009 |  |
|  | *Trichostrongyloidea* sp. | YES | FR | 7.10% | Pisanu et al. 2009 |  |
|  | *Trichuris* sp. | YES | FR | 9.50% | Pisanu et al. 2009 |  |
| *Muntiacus reevesi* | *Anaplasma phagocytophilum* | YES | UK | 1% | Duscher et al. 2020 |  |
|  | *Bovine Viral Diarrhoea Virus* (BVDV) | NO | IE |  | McKillen et al. 2017 | Can act as a reservoir. Preliminary study. |
|  | *Foot and Mouth Disease Virus* (FMDV) | NO | UK |  | Gibbs et al. 1975 | Samples analysed were already infected. |
|  | *Ixodes ricinus* |  | UK |  | GB Non-Native Species Secretariat, 2011 |  |
|  | *Mycobacterium bovis* | YES | UK |  | Ward & Smith, 2012 | Can act as a host. |
| *Myocastor coypus* | *Cryptosporidium* spp. | YES | IT | 0% | Zanzani et al. 2016 |  |
|  | *Cryptosporidium* spp. | YES | CZ | 0% | Kellnerová et al. 2017 |  |
|  | *Eimeria coypi* | NO | CZ | 37% | Nechybová et al. 2018 | Faecal analysis of farm-bred animals. |
| Species | Pathogen | Zoonotic | Country | Prevalence | Reference | Notes |
| *Myocastor coypus* | *Eimeria coypi* | NO | CZ | 60% | Nechybová et al. 2018 | Faecal analysis of wild animals. |
|  | *Eimeria coypi* | NO | IT | 86.30% | Zanzani et al. 2016 |  |
|  | *Eimeria myopotami* | NO | CZ | 5% | Nechybová et al. 2018 | Faecal analysis of farm-bred animals. |
|  | *Eimeria nutriae* | NO | CZ | 45% | Nechybová et al. 2018 | Faecal analysis of wild animals. |
|  | *Eimeria nutriae* | NO | CZ | 23% | Nechybová et al. 2018 | Faecal analysis of farm-bred animals. |
|  | *Eimeria seideli* | NO | CZ | 26% | Nechybová et al. 2018 | Faecal analysis of farm-bred animals. |
|  | *Eimeria seideli* | NO | IT | 6.80% | Zanzani et al. 2016 |  |
|  | *Escherichia coli* | YES | IT | 4.50% | Zanzani et al. 2016 |  |
|  | *Francisella tularensis* | YES | DE | 0% | Schulze et al. 2016 |  |
|  | *Giardia duodenalis (Giardia lamblia)* | YES | IT | 0% | Zanzani et al. 2016 |  |
|  | *Hepatitis E Virus* (HEV) | YES | IT | 0% | Serracca et al. 2015 |  |
|  | *Leptospira interrogans* | YES | IT | 44.90% | Zanzani et al. 2016 | Antibodies. Humans are accidental hosts. |
|  | *Leptospira* spp. | YES | IT | 32.90% | Bertelloni et al. 2019 |  |
|  | *Leptospira* spp. | YES | IT | 44.90% | Zanzani et al. 2016 |  |
|  | *Leptospira* spp. | YES | IT | 27.90% | Fratini et al. 2015 | Antibodies. Prevalence 9.8% by PCR, 0% by bacteriological examination. |
|  | *Leptospira* spp. | YES | FR | 64%-76% | Vein et al. 2014 | Antibodies. |
|  | *Leptospira* spp. | YES | FR | 42% | Ayral et al. 2020 | Antibodies. |
|  | *Leptospira* spp. | YES | FR | 16.50%-66% | Michel et al. 2001 | Antibodies. |
|  | *Salmonella* spp. | YES | IT | 0% | Zanzani et al. 2016 |  |
|  | *Staphylococcus aureus* | YES | IT | 10.10% | Zanzani et al. 2016 |  |
|  | *Streptococcus* spp. | YES | IT | 3.40% | Zanzani et al. 2016 |  |
|  | *Strongyloides myopotami* | YES | CZ | 25% | Nechybová et al. 2018 | Necropsy on farm-bred animals. |
|  | *Strongyloides myopotami* | YES | IT | 63.40% | Zanzani et al. 2016 |  |
|  | *Strongyloides* sp. | YES | CZ | 30% | Nechybová et al. 2018 | Faecal analysis of wild animals. |
|  | *Strongyloides* sp. | YES | CZ | 11.50% | Nechybová et al. 2018 | Faecal analysis of farm-bred animals. |
| Species | Pathogen | Zoonotic | Country | Prevalence | Reference | Notes |
| *Myocastor coypus* | *Toxoplasma gondii* | YES | IT | 28.9% | Zanzani et al. 2016 | Antibodies. |
|  | *Toxoplasma gondii* | YES | IT | 59.40% | Nardoni et al. 2011 | Antibodies. Prevalence 52.2% by PCR. |
|  | *Trichostrongylus duretteae* | NO | IT | 28.10% | Zanzani et al. 2016 |  |
|  | *Trichostrongylus* sp. | YES | CZ | 4% | Nechybová et al. 2018 | Faecal analysis of farm-bred animals. |
|  | *Trichuris myocastoris* |  | CZ | 40% | Nechybová et al. 2018 | Necropsy on farm-bred animals. |
|  | *Trichuris* sp. | YES | CZ | 5% | Nechybová et al. 2018 | Faecal analysis of wild animals. |
|  | *Trichuris* sp. | YES | CZ | 57% | Nechybová et al. 2018 | Faecal analysis of farm-bred animals. |
| *Neovison vison* | *Aelurostrongylus* spp. | NO | ES | 2% | Martínez-Rondán et al. 2017 |  |
|  | *Alaria alata* | YES | LT | 7.60% | Nugaraitė et al. 2018 | Mesocercariae. |
|  | *Aleutian Disease Virus* (ADV) | NO | ES |  | Mañas et al. 2001 | ADV DNA was detected by PCR in 28.57% of the carcasses tested. |
|  | *Angiostrongylus daskalovi* | NO | ES | 6% | Martínez-Rondán et al. 2017 |  |
|  | *Angiostrongylus vasorum* | NO | DK | 0.80% | Lemming et al. 2020 |  |
|  | *Aonchotheca annulosa* | NO | ES | 8% | Martínez-Rondán et al. 2017 |  |
|  | *Aonchotheca putorii* | YES | ES | 54% | Martínez-Rondán et al. 2017 |  |
|  | *Aonchotheca putorii* | YES | LT | 33.30%-50% | Nugaraitė et al. 2018 |  |
|  | *Canine ParvoVirus* (CPV) | NO | PT | 0% | Miranda et al. 2017 |  |
|  | *Capillaria plica (Pearsonema plica)* | NO | DK | 0% | Petersen et al. 2018b |  |
|  | *Crenosoma melesi* |  | ES | 10% | Martínez-Rondán et al. 2017 |  |
|  | *Crenosoma schachmatovae* |  | LT | 10.20%-15% | Nugaraitė et al. 2018 |  |
|  | *Crenosoma vulpis* | NO | DK | 5.70% | Lemming et al. 2020 |  |
|  | *Cryptosporidium* spp. | YES | CZ | 1% | Kellnerová et al. 2017 |  |
|  | *Cystoisospora* spp. | YES | DK | 11% | Petersen et al. 2020 |  |
|  | *Echinococcus* spp. | YES | PL | 14.20% | Kołodziej-Sobocińska et al. 2020 |  |
|  | *Ehrlichia canis* | YES | ES | 0% | Criado-Fornelio et al. 2018 |  |
| Species | Pathogen | Zoonotic | Country | Prevalence | Reference | Notes |
| *Neovison vison* | *Eucoleus aerophilus* | YES | LT | 10%-15.30% | Nugaraitė et al. 2018 |  |
|  | *Francisella tularensis* | YES | DE | 0% | Schulze et al. 2016 |  |
|  | *Hepatozoon* spp. | NO | ES | 0% | Criado-Fornelio et al. 2018 |  |
|  | *Influenza A Viruses* (IAV) | YES | ES | 2.20% | Gholipour et al. 2017 |  |
|  | *Isthmiophora melis* | NO | LT | 75% | Nugaraitė et al. 2017 |  |
|  | *Isthmiophora melis* | NO | LT | 70%-77% | Nugaraitė et al. 2018 |  |
|  | *Mesocestoides* spp. | YES | LT | 5%-7.60% | Nugaraitė et al. 2018 |  |
|  | *Molineus patens* | NO | LT | 12.80%-20% | Nugaraitė et al. 2018 |  |
|  | *Molineus patens* | NO | ES | 68% | Martínez-Rondán et al. 2017 |  |
|  | *Pseudamphistomum truncatum* | YES | LT | 17.90%-30% | Nugaraitė et al. 2018 |  |
|  | *Sarcosystis lutrae* | NO | LT | 13.60% | Prakas et al. 2018 |  |
|  | *SARS-CoV-2* | YES | NL | 19.40% | Oreshkova et al. 2020 | Dead mink positive for viral RNA. Prevalence 100% of the throat swabs of dead animals. |
|  | *Skrjabingylus nasicola* | NO | DE | 53.30% | Heddergott et al. 2016 |  |
|  | *Staphylococcus aureus methicillin-resistant* (LA-MRSA) | YES | DK | 34%-40% | Hansen et al. 2017 |  |
|  | *Strigea strigis* |  | LT | 28.20%-30% | Nugaraitė et al. 2018 | Metacercariae. |
|  | *Taenia martis* | YES | LT | 2.50% | Nugaraitė et al. 2018 |  |
|  | *Toxocara* spp. | YES | PL | 21.70% | Kołodziej-Sobocińska et al. 2020 |  |
|  | *Toxoplasma gondii* | YES | PL | 25% | Sroka et al. 2019 |  |
|  | *Toxoplasma gondii* | YES | ES | 78.80% | Ribas et al. 2018 |  |
|  | *Toxoplasma gondii* | YES | ES | 0% | Criado-Fornelio et al. 2018 |  |
|  | *Trichinella* spp. | YES | PL | 3.30% | Hurníková et al. 2016 |  |
|  | *Troglotrema acutum* |  | ES | 2% | Martínez-Rondán et al. 2017 |  |
|  | Unidentified trematode |  | ES | 2% | Martínez-Rondán et al. 2017 |  |
|  |  |  |  |  |  |  |
| Species | Pathogen | Zoonotic | Country | Prevalence | Reference | Notes |
| *Nyctereutes procyonoides* | *Aelurostrongylus abstrusus* | NO | DK | 0% | Lemming et al. 2020 |  |
|  | *Alaria alata* | YES | AT | 30% | Duscher et al. 2017 |  |
|  | *Alaria alata* | YES | EE | 13.30% | Laurimaa et al. 2016 | Metacercariae. |
|  | *Alaria alata* | YES | EE | 68.30% | Laurimaa et al. 2016 |  |
|  | *Alaria alata* | YES | PL | 94.30% | Karamon et al. 2016 |  |
|  | Anaplasmataceae | YES | AT | 0% | Duscher et al. 2017 |  |
|  | *Angiostrongylus vasorum* | NO | EE | 1.30% | Laurimaa et al. 2016 |  |
|  | *Angiostrongylus vasorum* | NO | DK | 3.20% | Lemming et al. 2020 |  |
|  | *Aonchotheca putorii* | YES | EE | 3.60% | Laurimaa et al. 2016 |  |
|  | *Apophallus* spp. | YES | PL | 15.10% | Karamon et al. 2016 |  |
|  | *Babesia cf microti* | YES | AT | 62.50% | Duscher et al. 2017 |  |
|  | *Borrelia* spp. | YES | PL | 25% | Wodecka et al. 2016 |  |
|  | *Candidatus Neoerlichia* sp. | YES | PL | 30% | Hildebrand et al. 2018 |  |
|  | *Capillaria aerophila (Eucoleus aerophilus)* | YES | DK | 1.90% | Lemming et al. 2020 |  |
|  | *Capillaria plica (Pearsonema plica)* | NO | DK | 0.50% | Petersen et al. 2018b |  |
|  | *Chlamydia* spp. | YES | UA | 0% | Ksyonz et al. 2019 |  |
|  | *Crenosoma vulpis* | NO | EE | 15% | Laurimaa et al. 2016 |  |
|  | *Crenosoma vulpis* | NO | DK | 5.30% | Lemming et al. 2020 |  |
|  | *Dipylidium caninum* | NO | AT | 20% | Duscher et al. 2017 |  |
|  | *Echinococcus multilocularis* | YES | AT | 10% | Duscher et al. 2017 |  |
|  | *Echinococcus multilocularis* | YES | DK | 0.70% | Petersen et al. 2018a |  |
|  | *Echinococcus multilocularis* | YES | EE | 1.60% | Laurimaa et al. 2016 |  |
| Species | Pathogen | Zoonotic | Country | Prevalence | Reference | Notes |
| *Nyctereutes procyonoides* | *Echinococcus multilocularis* | YES | NL | 11.10% | Maas et al. 2016 | PCR. |
|  | *Echinococcus multilocularis* | YES | DK | 0% | Oksanen et al. 2016 | Pooled prevalence from Enemark, 2013; Al-Sabi et al. 2013; EFSA, 2015. |
|  | *Echinococcus multilocularis* | YES | DE | 2.50% | Oksanen et al. 2016 | Pooled prevalence from Thiess et al. 2001; Thiess, 2004; Schwarz et al. 2011. |
|  | *Echinococcus multilocularis* | YES | NL | 0% | EFSA, 2015 |  |
|  | *Echinococcus multilocularis* | YES | FI | 0% | Oksanen et al. 2016 | Pooled prevalence from EFSA, 2013, 2014, 2015. |
|  | *Echinococcus multilocularis* | YES | PL | 10.40% | Oksanen et al. 2016 | Pooled prevalence from Machnicka-Rowińska et al. 2002; Machnicka et al. 2003; EFSA, 2015. |
|  | *Echinococcus multilocularis* | YES | PL | 0% | Karamon et al. 2016 |  |
|  | *Echinococcus multilocularis* | YES | SE | 0% | Wahlström et al. 2011 |  |
|  | *Echinococcus multilocularis* | YES | SK | 28% | Oksanen et al. 2016 | Pooled prevalence from Letková et al. 2008; Hurníková et al. 2009; EFSA, 2015. |
|  | *Echinococcus multilocularis* | YES | LV | 8.10% | Bagrade et al. 2016 |  |
|  | *Echinococcus multilocularis* | YES | LV | 21% | Bagrade et al. 2008 |  |
|  | *Echinococcus multilocularis* | YES | LT | 8.20% | Bružinskaitė-Schmidhalter et al. 2012 |  |
|  | *Echinococcus multilocularis* | YES | UA | 0% | Kornyushin et al. 2011 |  |
|  | Echinostomatidae | YES | PL | 18.90% | Karamon et al. 2016 |  |
|  | *Eucoleus aerophilus* | YES | EE | 30% | Laurimaa et al. 2016 |  |
|  | *Francisella tularensis* | YES | DE | 16.70% | Schulze et al. 2016 |  |
|  | *Hepatitis E Virus (HEV)* | YES | DE | 34.30% | Dahnert et al. 2018 |  |
|  | Hookworms | YES | PL | 83% | Karamon et al. 2016 |  |
|  | *Isthmiophora melis* | NO | AT | 20% | Duscher et al. 2017 |  |
|  |  |  |  |  |  |  |
| Species | Pathogen | Zoonotic | Country | Prevalence | Reference | Notes |
| *Nyctereutes procyonoides* | *Isthmiophora melis* | NO | EE | 6% | Laurimaa et al. 2016 |  |
|  | *Ixodes ricinus* |  | PL |  | Wodecka et al. 2016 | Raccoon dogs harbor seven-fold more ticks than badgers. |
|  | *Mesocestoides* spp. | YES | AT | 40% | Duscher et al. 2017 |  |
|  | *Mesocestoides* spp. | YES | EE | 21.30% | Laurimaa et al. 2016 | *M. lineatus, M. litteratus* |
|  | *Metorchis bilis* | YES | EE | 19.50% | Laurimaa et al. 2016 |  |
|  | *Molineus patens* | NO | EE | 13.70% | Laurimaa et al. 2016 |  |
|  | *Molineus* spp. | NO | AT | 30% | Duscher et al. 2017 |  |
|  | *Molineus* spp. | NO | PL | 41.50% | Karamon et al. 2016 |  |
|  | *Pearsonema plica* | NO | EE | 10.80% | Laurimaa et al. 2016 |  |
|  | *Plagiorchis elegans* | NO | EE | 0.80% | Laurimaa et al. 2016 |  |
|  | *Pygidiopsis summa* | YES | DK | 3% | Al-Sabi et al. 2013 |  |
|  | *Taenia policantha* | NO | EE | 8.40% | Laurimaa et al. 2016 |  |
|  | *Taenia* spp. | YES | AT | 20% | Duscher et al. 2017 |  |
|  | *Tick-Borne Encephalitis Virus* (TBEV) | YES | FI |  | Uusitalo et al. 2020 | Has a role in the cycle. |
|  | *Toxocara canis* | YES | AT | 20% | Duscher et al. 2017 |  |
|  | *Toxocara leonina* | YES | AT | 10% | Duscher et al. 2017 |  |
|  | *Toxocara* spp. | YES | EE | 8% | Laurimaa et al. 2016 | *T. canis, T. leonina* |
|  | *Toxocara* spp. | YES | PL | 15.10% | Karamon et al. 2016 |  |
|  | *Toxoplasma gondii* | YES | PL | 7.70% | Sroka et al. 2019 |  |
|  | *Trichinella* spp. | YES | AT | 0% | Duscher et al. 2017 |  |
|  | *Trichinella* spp. | YES | EE | 57.50% | Kärssin et al. 2017 |  |
|  | *Trichinella* spp. | YES | NL | 11.10% | Maas et al. 2016 |  |
|  | *Trichinella* spp. | YES | PL | 39.80% | Cybulska et al. 2019 |  |
|  | *Uncinaria stenocephala* | YES | AT | 40% | Duscher et al. 2017 |  |
|  | *Uncinaria stenocephala* | YES | EE | 97.60% | Laurimaa et al. 2016 |  |
|  | Unidentified lungworm |  | DK | 0.80% | Lemming et al. 2020 |  |
|  |  |  |  |  |  |  |
| Species | Pathogen | Zoonotic | Country | Prevalence | Reference | Notes |
| *Ondatra zibethicus* | *Bartonella* spp. | YES | BE |  | Krügel et al. 2020 | Detection in a by-caught specimen. |
|  | *Chlamydia* spp. | YES | UA | 33.30% | Ksyonz et al. 2019 |  |
|  | *Cryptosporidium* spp. | YES | DE |  | Petri et al. 1997 | Muskrat can contaminate waters. |
|  | *Echinococcus multilocularis* | YES | BE | 11.20% | Hanosset et al. 2008 |  |
|  | *Echinococcus multilocularis* | YES | NL | 0.10% | Borgsteede et al. 2003 |  |
|  | *Francisella tularensis* | YES | DE | 0% | Schulze et al. 2016 |  |
|  | *Giardia duodenalis (Giardia lamblia)* | YES | RO | 100% | Adriana et al. 2016 | One sample analysed. |
|  | *Leptospira spp.* | YES | DE | 5.90% | Hurd et al. 2017 |  |
|  | *Toxoplasma gondii* | YES | PL | 6.30% | Sroka et al. 2019 |  |
|  | *Yersinia pestis* | YES |  |  | Anon., 1940 | Appears to be susceptible to plague. |
| *Procyon lotor* | *Acanthocephala* | YES | PL | 1.90% | Karamon et al. 2014 |  |
|  | *Alaria alata* | YES | AT | 0% | Duscher et al. 2017 |  |
|  | *Alaria alata* | YES | DE | 33.30% | Rentería-Solís et al. 2013 |  |
|  | *Anaplasma phagocytophilum* | YES | PL | 0.80% | Hildebrand et al. 2018 |  |
|  | Anaplasmataceae | YES | AT | 0% | Duscher et al. 2017 |  |
|  | *Ancylostoma* spp. | YES | PL | 4.40% | Popiołek et al. 2011 |  |
|  | *Babesia cf microti* | YES | AT | 0% | Duscher et al. 2017 |  |
|  | *Baylisascaris procyonis* | YES | DE | 43.60% | Heddergott et al. 2020b |  |
|  | *Baylisascaris procyonis* | YES | DE | 76.20% | Rentería-Solís et al. 2018 |  |
|  | *Baylisascaris procyonis* | YES | DE | 39% | Winter, 2005 |  |
|  | *Baylisascaris procyonis* | YES | DE | 71.40% | Gey, 1998 |  |
|  | *Baylisascaris procyonis* | YES | DE | 80% | Hohmann et al. 2002 |  |
|  | *Baylisascaris procyonis* | YES | DK | 11% | Al-Sabi et al. 2016 |  |
|  | *Baylisascaris procyonis* | YES | PL | 3.30% | Popiołek et al. 2011 |  |
|  | *Baylisascaris procyonis* | YES | PL | 1.90% | Karamon et al. 2014 |  |
| Species | Pathogen | Zoonotic | Country | Prevalence | Reference | Notes |
| *Procyon lotor* | *Baylisascaris procyonis* | YES | PL | 3.70% | Bartoszewicz et al. 2008 |  |
|  | *Canine Adenovirus 1* (CAdV-1) | NO | DE | 0% | Schulze et al. 2019 |  |
|  | *Canine Adenovirus 1* (CAdV-1) | NO | DE | 0% | Hechinger et al. 2017 |  |
|  | *Canine Adenovirus 1* (CAdV-2) | NO | DE | 0% | Schulze et al. 2019 |  |
|  | *Canine Distemper Virus* (CDV) | NO | DE | 10.80% | Wibbelt et al. 2008 |  |
|  | *Canine Distemper Virus* (CDV) | NO | DE | 46% | Hechinger et al. 2017 |  |
|  | *Canine Distemper Virus* (CDV) | NO | DE | 76.30% | Rentería-Solís et al. 2014b |  |
|  | *Capillaria* spp. | YES | PL | 25.50% | Karamon et al. 2014 |  |
|  | Capillaridae | YES | PL | 33.30% | Popiołek et al. 2011 |  |
|  | *Cryptosporidium* spp. | YES | LU | 12.40% | Heddergott et al. 2020a |  |
|  | *Cryptosporidium* spp. | YES | PL | 34.70% | Leśniańska et al. 2016 |  |
|  | *Cryptosporidium* spp. | YES | DE | 34.70% | Leśniańska et al. 2016 |  |
|  | *Dipylidium caninum* | NO | AT | 0% | Duscher et al. 2017 |  |
|  | *Echinococcus multilocularis* | YES | AT | 0% | Duscher et al. 2017 |  |
|  | *Echinostoma* sp. | YES | PL | 2.20% | Popiołek et al. 2011 |  |
|  | Echinostomatidae | YES | PL | 34.50% | Karamon et al. 2014 |  |
|  | *Ehrlichia canis* | YES | ES | 2.60% | Criado-Fornelio et al. 2018 |  |
|  | *Eimeria* spp. | YES | DE | 1.50% | Gey, 1998 |  |
|  | *Eimeria* spp. | YES | DE | 1.80% | Winter, 2005 |  |
|  | *Enterocytozoon bieneusi* | YES | PL | 4.10% | Leśniańska et al. 2016 |  |
|  | *Francisella tularensis* | YES | DE | 0% | Schulze et al. 2016 |  |
|  | *Hepatitis E Virus (HEV)* | YES | DE | 53.80% | Dahnert et al. 2018 |  |
|  | *Hepatozoon canis* | NO | ES | 2.60% | Criado-Fornelio et al. 2018 |  |
| Species | Pathogen | Zoonotic | Country | Prevalence | Reference | Notes |
| *Procyon lotor* | *Isthmiophora melis* | NO | AT | 0% | Duscher et al. 2017 |  |
|  | *Leishmania infantum* | YES | ES | 0% | Risueño et al. 2018 |  |
|  | *Listeria* spp. | YES | PL | 7.10% | Nowakiewicz et al. 2016 |  |
|  | *Lyssavirus rabies* | YES | Europe |  | The Rabies Information System of the WHO Collaboration Centre for Rabies Surveillance and Research | 142 cases reported in Europe. http://rbe.fli.bund.de/ Default.aspx |
|  | *Mesocestoides* spp. | YES | AT | 0% | Duscher et al. 2017 |  |
|  | *Mesocestoides* spp. | YES | PL | 67.30% | Karamon et al. 2014 |  |
|  | *Molineus* spp. | NO | AT | 13% | Duscher et al. 2017 |  |
|  | *Neospora caninum* | NO | CZ | 17.60% | Kornacka et al. 2018 | Antibodies. Prevalence 0% by PCR. |
|  | *Neospora caninum* | NO | DE | 16.70% | Kornacka et al. 2018 | Antibodies. Prevalence 0% by PCR. |
|  | *Neospora caninum* | NO | PL | 13.30% | Kornacka et al. 2018 | Antibodies. Prevalence 0% by PCR. |
|  | *Placoconus lotoris* | NO | PL | 4.40% | Popiołek et al. 2011 |  |
|  | *Salmonella* spp. | YES | PL | 5.70% | Nowakiewicz et al. 2016 |  |
|  | *Sarcocystis* spp. | YES | DE | 4.40% | Stolte et al. 1996 |  |
|  | *Sarcocystis* spp. |  | DE |  | Rentería-Solís et al. 2014 | Cross-transmission of *S. scabiei* mites has been recorded. |
|  | *Spirocerca lupi* | NO | PL | 8.80% | Popiołek et al. 2011 |  |
|  | *Staphylococcus coagulase-positive* | YES | PL | 35.70% | Nowakiewicz et al. 2016 |  |
|  | *Strongyloides procyonis* | YES | PL | 14.80% | Bartoszewicz et al. 2008 |  |
|  | *Strongyloides procyonis* | YES | PL | 11% | Popiołek et al. 2011 |  |
|  | *Taenia* spp. | YES | AT | 0% | Duscher et al. 2017 |  |
|  | *Toxocara canis* | YES | AT | 0% | Duscher et al. 2017 |  |
|  | *Toxocara leonina* | YES | AT | 0% | Duscher et al. 2017 |  |
|  | *Toxoplasma gondii* | YES | CZ | 0% | Kornacka et al. 2018 | Antibodies. Prevalence 47.1% by PCR. |
|  | *Toxoplasma gondii* | YES | DE | 26% | Gey, 1998 |  |
|  | *Toxoplasma gondii* | YES | DE | 33.30% | Kornacka et al. 2018 | Antibodies. Prevalence 33.3% by PCR. |
|  | *Toxoplasma gondii* | YES | DE | 38.30% | Heddergot et al. 2017 | Antibodies. |
|  | *Toxoplasma gondii* | YES | LU | 19% | Heddergot et al. 2017 | Antibodies. |
| Species | Pathogen | Zoonotic | Country | Prevalence | Reference | Notes |
| *Procyon lotor* | *Toxoplasma gondii* | YES | PL | 13.10% | Sroka et al. 2019 |  |
|  | *Toxoplasma gondii* | YES | PL | 13.30% | Kornacka et al. 2018 | Antibodies. Prevalence 40% by PCR. |
|  | *Toxoplasma gondii* | YES | ES | 3.60% | Criado-Fornelio et al. 2018 |  |
|  | *Trichinella* spp. | YES | AT | 0% | Duscher et al. 2017 |  |
|  | *Trichinella* spp. | YES | CZ | 9.10% | Cybulska et al. 2018 |  |
|  | *Trichinella* spp. | YES | DE | 0% | Cybulska et al. 2018 |  |
|  | *Trichinella* spp. | YES | PL | 6% | Cybulska et al. 2018 |  |
|  | *Uncinaria stenocephala* | YES | AT | 0% | Duscher et al. 2017 |  |
|  | *Yersinia* spp. | YES | PL | 4.30% | Nowakiewicz et al. 2016 |  |
| *Sciurus carolinensis* | *Adenoviridae* | YES | IT | 0.90% | Romeo et al. 2014b |  |
|  | *Aonchotheca annulosa* | NO | IT | 1.50% | Romeo et al. 2014a |  |
|  | *Borrelia burgdoferi sensu lato* | YES | UK | 11.90% | Millins et al. 2015 |  |
|  | *Cryptosporidium* spp. | YES | IT | 3.70% | Prediger et al. 2017 |  |
|  | *Eimeria* spp. | YES | IT | 95.70% | Hofmannová et al. 2016 | Plus the successful introduction of *E. lancasterensis.* |
|  | Hymenolepididae | YES | IT | 0.40% | Romeo et al. 2014a |  |
|  | *Hymenolepis* spp. | YES | IT | 0% | d’Ovidio et al. 2015 |  |
|  | *Ljungan Virus* (LV) | YES | IT | 0% | Romeo et al. 2014b |  |
|  | *Mycobacterium leprae* | YES | IT | 0% | Schilling et al. 2019 |  |
|  | *Mycobacterium leprae* | YES | UK | 0% | Schilling et al. 2019 |  |
|  | Oxyurida | YES | IT | 0.90% | Romeo et al. 2014a |  |
|  | *Squirrelpox poxvirus* (SQPV) | NO | IE | 29% | Stritch et al. 2015 |  |
|  | *Squirrelpox poxvirus* (SQPV) | NO | IE | 25% | Collins et al. 2014 | Antibodies. Prevalence 10% by PCR. |
|  | Strongylida | YES | IT | 4.40% | Romeo et al. 2014a |  |
|  | *Strongyloides robustus* | NO | IT | 56.50% | Romeo et al. 2014a |  |
|  | *Tick-Borne Encephalitis Virus* (TBEV) | YES | IT | 1.90%-2.50% | Romeo et al. 2018 |  |
| Species | Pathogen | Zoonotic | Country | Prevalence | Reference | Notes |
| *Sciurus carolinensis* | *Trichostrongylus calcaratus* | NO | IT | 6.50% | Romeo et al. 2014a |  |
|  | *Trichostrongylus retortaeformis* | NO | IT | 0.80% | Romeo et al. 2014a |  |
|  | *Trichuris muris* | NO | IT | 4.20% | Romeo et al. 2014a |  |
|  | *Trypanoxyuris sciuri* | NO | IT | 2.30% | Romeo et al. 2014a |  |
|  | *Usutu Virus* (USUV) | YES | IT | 3.20%-3.80% | Romeo et al. 2018 |  |
|  | *West Nile Virus* (WNV) | YES | IT | 0.60% | Romeo et al. 2018 |  |

**List of the additional references for the pathogen studies (studies not directly included in the review).**

*Cervus nippon*

Dykova I, Blazek K (1972). Subcutaneous filariasis in red deer. *Acta Veterinaria* 41: 117–124.

Gibbs EPJ, Herniman KAJ, Lawman MJP (1975) Studies with foot-and-mouth disease virus in British deer (muntjac and sika): Clinical disease, recovery of virus and serological response. *Journal of Comparative Pathology* 85(3): 361–366

Mihalca AD, Păstrav IR, Sándor AD, Deak G, Gherman CM, Sarmaşi A et al. (2019) First report of the dog louse fly *Hippobosca longipennis* in Romania. *Medical and Veterinary Entomology* 33(4): 530–535.

Nechybová S, Vejl P, Hart V, Melounová M, Čílová D, Vašek J et al. (2018) Long-term occurrence of Trichuris species in wild ruminants in the Czech Republic. Parasitology Research 117(6): 1699–1708.

Rudaitytė-Lukošienė E, Prakas P, Butkauskas, D, Kutkienė L, Vepštaitė-Monstavičė I, Servienė E (2018) Morphological and molecular identification of *Sarcocystis* spp. from the sika deer (*Cervus nippon*), including two new species *Sarcocystis frondea* and *Sarcocystis nipponi*. *Parasitology Research* 117(5): 1305–1315.

*Myocastor coypus*

Michel V, Ruveon-Clouet N, Menard A, Sonrier C, Fillonneau C, Rakotovao F et al. (2001) Role of the coypu (*Myocastor coypus*) in the epidemiology of leptospirosis in domestic animals and humans in France. *European Journal of Epidemiology* 17: 111–121.

*Neovison vison*

Hansen JE, Larsen AR, Skov RL, Chriél M, Larsen G, Angen Ø (2017). Livestock-associated methicillin-resistant *Staphylococcus aureus* is widespread in farmed mink (*Neovison vison*). *Veterinary Microbiology* 207: 44–49.

Mañas S, Carlos Ceña J, Ruiz-Olmo J, Palazón S, Domingo M, Wolfinbarger JB et al. (2001) Aleutian mink disease parvovirus in wild riparian carnivores in Spain. *Journal of Wildlife Diseases* 37(1): 138–144.

Martínez-Rondán F, Ruiz de Ybañez R, Tizzani P, López-Beceiro A, Fidalgo L, Martínez-Carrasco Pleite C (2017) The American mink (*Neovison vison*) is a competent host for native European parasites. *Veterinary Parasitology* 247: 93–99.

*Nyctereutes procyonoides*

Al-Sabi MNS, Chriél M,Hammer Jensen T, Larsen Enemark H (2013). Endoparasites of the raccoon dog (*Nyctereutes procyonoides*) and the red fox (*Vulpes vulpes*) in Denmark 2009–2012 – A comparative study. *International Journal for Parasitology: Parasites and Wildlife* 2: 144–151.

Bagrade G, Snabel V, Romig T, Ozolins J, Huettner M, Miterpáková M et al. (2008) *Echinococcus multilocularis* is a frequent parasite of red foxes (*Vulpes vulpes*) in Latvia. *Helminthologia* 45: 157–161.

Bružinskaitė-Schmidhalter R, Šarkūnas M, Malakauskas A, Mathis A, Torgerson PR, Deplazes P (2012) Helminths of red foxes (*Vulpes vulpes*) and raccoon dogs (*Nyctereutes procyonoides*) in Lithuania. *Parasitology* 139: 120–127.

Dähnert L, Conraths F, Reimer N, Groschup M, Eiden M (2018) Molecular and serological surveillance of Hepatitis E virus in wild and domestic carnivores in Brandenburg, Germany. *Transboundary and Emerging Diseases* 65(5).

EFSA (2015) Scientific opinion – Update on oral vaccination of foxes and raccoon dogs against rabies. *EFSA Journal* 13: 70.

Kornyushin VV, Malyshko EI, Malega AM (2011) The Helminths of wild predatory mammals of Ukraine. Cestodes. *Vestnik Zoologii* 45: 4–11.

Wahlström H, Lindberg A, Lindh J, Wallensten A, Lindqvist R, Plym-Forshell L et al. (2012) Investigations and actions taken during 2011 due to the first finding of *Echinococcus multilocularis* in Sweden. *Eurosurveillance* 17: 1–7.

*Ondatra zibethicus*

Anon, 1940. The possible role of the muskrat (*Ondatra zibethica* L.) in the epidemiology of plague. *Vestnik Mikrobiologii, Epidemiologii i Parazitologii*, 19(2).

Borgsteede FHM, van der Tibben JH, Giessen JWB (2003). The muskrat (*Ondatra zibethicus*) as intermediate host of cestodes in the Netherlands. *Veterinary Parasitology* 117: 29–36.

Hanosset R, Saegerman C, Adant S, Massart L, Losson B (2008) *Echinococcus multilocularis* in Belgium: prevalence in red foxes (*Vulpes vulpes*) and in different species of potential intermediate hosts. *Veterinary Parasitology* 151(2/4): 212–217.

Petri C, Karanis P, Renoth S (1997) Cryptosporidium infections in muskrat (*Ondatra zibethica*). *Parasite* 4(4): 369–371.

*Procyon lotor*

Bartoszewicz M, Okarma H, Zalewski A, Szczesna J (2008). Ecology of the raccoon (*Procyon lotor*) from western Poland. *Annales Zoologici Fennici* 45(4): 291–298.

Dähnert L, Conraths FJ, Reimer N, Groschup MH, Eiden M (2018) Molecular and serological surveillance of hepatitis E virus in wild and domestic carnivores in Brandenburg, Germany. *Transboundary Emerging Diseases* 65: 1377–1380.

Gey AB (1998). Endoparasite fauna of the raccoon (*Procyon lotor*) in Hesse, Germany. PhD thesis, Justus-Liebieg University Giesen, Germany. [in German with English summary].

Hohmann U, Voig S, Andreas U (2002) Raccoons take the offensive. A current assessment. In: *Biologische Invasionen*, edited by Kowarik I, Starfinger U, *Neobiota* 1: 191–192.

Stolte M, Odening K, Walter G, Bockhardt I (1996) The raccoon as intermediate host of three Sarcocystis species in Europe. *Comparative parasitology* 63(1): 145–149.

Wibbelt G, Speck S, Fickel J, Köhnemann B, Michler F.-U. (2008) Outbreak of canine distemper in raccoons (*Procyon lotor*) in Germany. 8th Conference of the Wildlife Disease Association, Rovinj/Kroatien, pp 22.

Winter M, Stubbe M, Heidecke D (2005) Zur Ökologie des Waschbären (*Procyon lotor* L., 1758) in Sachsen-Anhalt. *Beitr Jagd Wildforsch* 30: 303–322. [in German].
